# Loneliness and desire for thinness explain sex differences in adolescent internalizing problems

**DOI:** 10.1038/s41562-026-02578-2

**Published:** 2026-09-15

**Authors:** Zui C. Narita, Gemma Knowles, Susan M. Sawyer, Syudo Yamasaki, Darío Moreno-Agostino, Jordan DeVylder, Junko Niimura, Naomi Nakajima, Satoshi Yamaguchi, Mariko Hosozawa, Miharu Nakanishi, Yutaka Sawai, Daiki Nagaoka, Ryo Sasaki, Rui Zhou, Naho Yamane, Jacqueline Cosse, Daniel Stanyon, Haruka Goto, Riki Tanaka, Akito Uno, Satoshi Usami, Shuntaro Ando, Mariko Hiraiwa-Hasegawa, Toshi A. Furukawa, Tomohiro Shinozaki, Kiyoto Kasai, Vikram Patel, Atsushi Nishida, the Bridging Divides Young Persons Advisory Group, Yumi Nakaegawa, Yayoi Muramatsu, Riko Suzuki, Nagisa Katoono, Keigo Oyamada, Rin Kataoka, Koutarou Takada

**Affiliations:** Tokyo Teen Cohort, Tokyo, Japan; 1 Department of Behavioral Medicine, National Institute of Mental Health, National Center of Neurology and Psychiatry, Tokyo, Japan; 2 Unit for Mental Health Promotion, Research Center for Social Science & Medicine, Tokyo Metropolitan Institute of Medical Science, Tokyo, Japan; 3 Economic and Social Research Council (ESRC) Centre for Society and Mental Health and Health Service and Population Research Department, Institute of Psychiatry, Psychology, and Neuroscience, King’s College London, London, UK; 4 Department of Paediatrics, The University of Melbourne, Melbourne, Australia; 5 Centre for Adolescent Health, Royal Children’s Hospital, Melbourne, Australia; 6 Murdoch Children’s Research Institute, Melbourne, Australia; 7 Centre for Longitudinal Studies, UCL Social Research Institute, University College London, UK; 8 ESRC Centre for Society and Mental Health, King’s College London, UK; 9 Silver School of Social Work, New York University, New York City, NY, USA; 10 Institute for Global Health Policy Research, Bureau of Global Health Cooperation, Japan Institute for Health Security; 11 Department of Psychiatric Nursing, Graduate School of Medicine, Tohoku University, Miyagi, Japan; 12 Department of Public Health, Juntendo University Graduate School of Medicine, Tokyo, Japan; 13 Department of Global Business for Health, University College London, London, UK; 14 Department of Neuropsychiatry, Graduate School of Medicine, The University of Tokyo, Tokyo, Japan; 15 Graduate School of Education, The University of Tokyo, Tokyo, Japan; and Center for Research and Development on Transition from Secondary to Higher Education, The University of Tokyo, Tokyo, Japan; 16 School of Advanced Science, SOKENDAI (Graduate University for Advanced Studies), Kanagawa, Japan; 17 Department of Health Promotion and Human Behavior, Kyoto University Graduate School of Medicine / School of Public Health, Kyoto, Japan; 18 Interfaculty Initiative in Information Studies and Department of Biostatistics, School of Public Health, Graduate School of Medicine, The University of Tokyo, Tokyo, Japan; 19 The International Research Center for Neurointelligence (WPI-IRCN), University of Tokyo Institutes of Advanced Study, Tokyo, Japan; 20 Department of Global Health and Social Medicine, Harvard Medical School, Boston, MA, USA

## Abstract

Girls are at higher risk of internalizing problems than boys. This disparity has widened in recent decades, but the underlying mechanisms remain understudied. We analyzed data from 3,171 adolescents (46.9% girls) within the Tokyo Teen Cohort, assessed at ages 12, 14, and 16. At age 16, girls showed higher prevalence of internalizing problems than boys: depressive symptoms (15.9% vs. 8.2%) and suicidal ideation (29.8% vs. 18.1%). Co-production with young people suggested loneliness and desire for thinness as candidate pathways. Using the mediational g-formula, we estimated that the effect through these factors at age 14 relative to the total effect of female sex was 34.7% for depressive symptoms and 46.3% for suicidal ideation at age 16. These findings suggest that social intermediates may contribute to the higher prevalence of internalizing problems among girls in this cohort, highlighting potential targets for interventions and policies informed by young people.

## Introduction

A well-established body of research shows that adult women are at higher risk of internalizing problems than men^1–3^. Understanding how and when these sex differences emerge is important for identifying intervention points. Adolescent girls are experiencing worsening psychological symptoms across a diverse range of countries and cultural settings^4^. An international study showed that this disparity is driven by worsening symptoms among girls rather than improvements in boys’ mental health^4^. In Japan, suicide deaths among girls have, for the first time, exceeded those among boys in 2024, a reversal of the longstanding pattern of boys accounting for far more suicide deaths^5^. Recent longitudinal evidence from Japan and the UK similarly shows that depressive symptoms increased year on year among girls, although the effects of age and calendar year could not be separated^6^. Another recent study from Australia showed that depressive and anxiety symptoms increased across adolescence for both sexes, but that the increase in prevalence was steeper in girls^7^. Adolescence may be a key period in which sex differences in internalizing problems begin to emerge and intensify, hence our call for greater attention to the mechanisms underlying this pattern in girls^8^. The present study builds directly on that call by investigating modifiable pathways, selected with and by young people, that may contribute to this pattern.

Disparities research has highlighted the importance of investigating the modifiable pathways associated with health outcomes^9–11^. Causal inference frameworks show that variables such as sex or race can be examined in analyses that focus on intervenable mediators through pathways leading to health outcomes^9,12^. Identifying such mediators allows researchers to assess the extent to which observed disparities are explained by specific mechanisms^13,14^. These mediators, including structural and social factors, represent potential targets for intervention; modifying them may help improve mental health outcomes among girls^13,14^.

Adolescence is a profound time of biopsychosocial change. While internalizing problems may emerge during this period, the perspectives of young people are often underrepresented in mental health research, which risks limiting practical relevance. Co-production provides a structured approach to address this limitation by integrating young people’s lived experiences into the design and interpretation of research^15–19^. In this study, structured discussions and workshops with young people were held to elicit their views on potential contributors to the higher prevalence of internalizing problems among girls. Undertaken as part of the Bridging Divides project (‘*Kakinemu*’), these co-produced insights informed the selection of mechanisms for analysis, ensuring that the research addressed factors recognized by young people as salient to their actual lives. Embedding co-production alongside causal inference analyses supports the scientific relevance and applicability of the findings, increasing the potential to identify modifiable pathways that can inform effective strategies^15^.

**Fig. 1:**
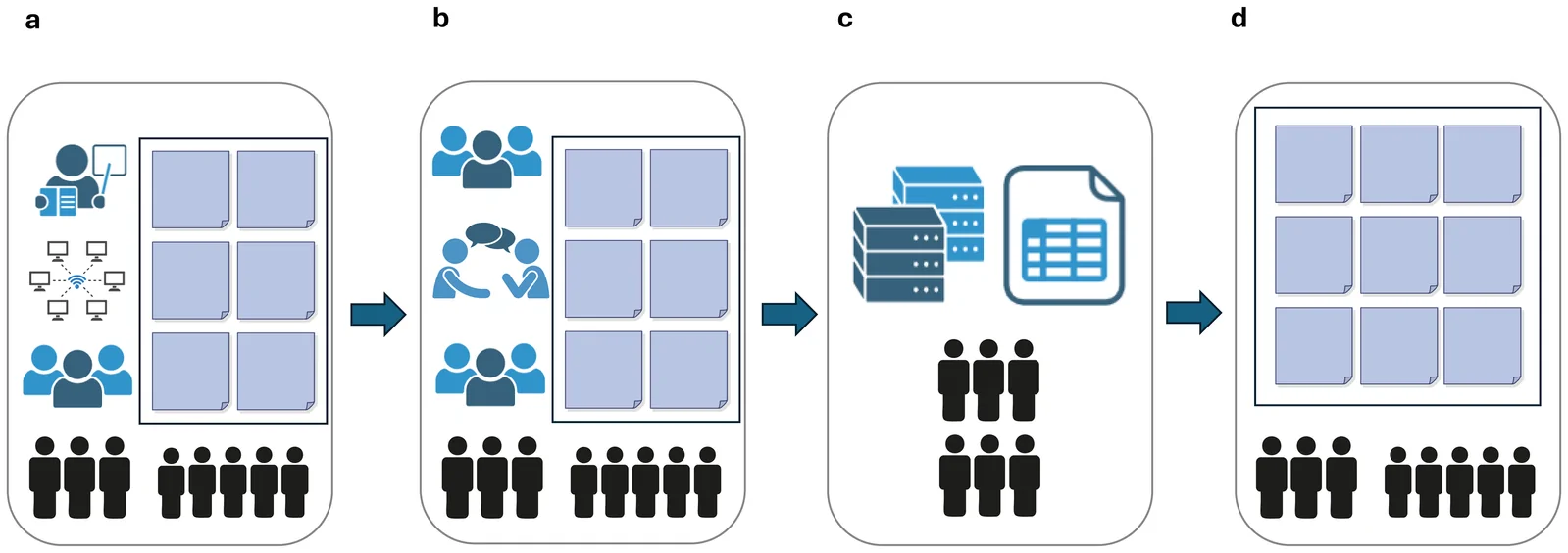
Co-production process involving young people and researchers. **a**, Early involvement of young people (beginning December 2022) during the interpretation of previous analyses on gender differences in adolescent mental health, where young people contributed ideas and an initial mindmap was drawn to inform subsequent study design and questions; development of the research proposal through online and offline collaboration and workshops with young people (up to April 2025) using a sticky note wall approach to collect, discuss, and cluster ideas on explanations for the higher prevalence of internalizing problems in girls. **b**, Structured discussion sessions (April 2025) bringing together young people and researchers; examination of multiple sticky note walls; and concurrent discussions among researchers on appropriate methods to examine pathways from female sex to internalizing problems. **c**, Review by researchers to evaluate the feasibility of the proposed mediators, considering data availability in the Tokyo Teen Cohort and alignment with the study’s causal framework. **d**, Follow-up joint meeting (September 2025) using a sticky note wall approach to further discuss and refine the potential mediators, allowing participants to post, cluster, and discuss ideas about why and how the selected mediators might matter in the context of the higher prevalence of internalizing problems in girls, ensuring that the final analysis addressed questions of direct relevance to young people while maintaining methodological rigor. Created in BioRender. Narita, Z. (2026) https://BioRender.com/ik10pin

**Fig. 2:**
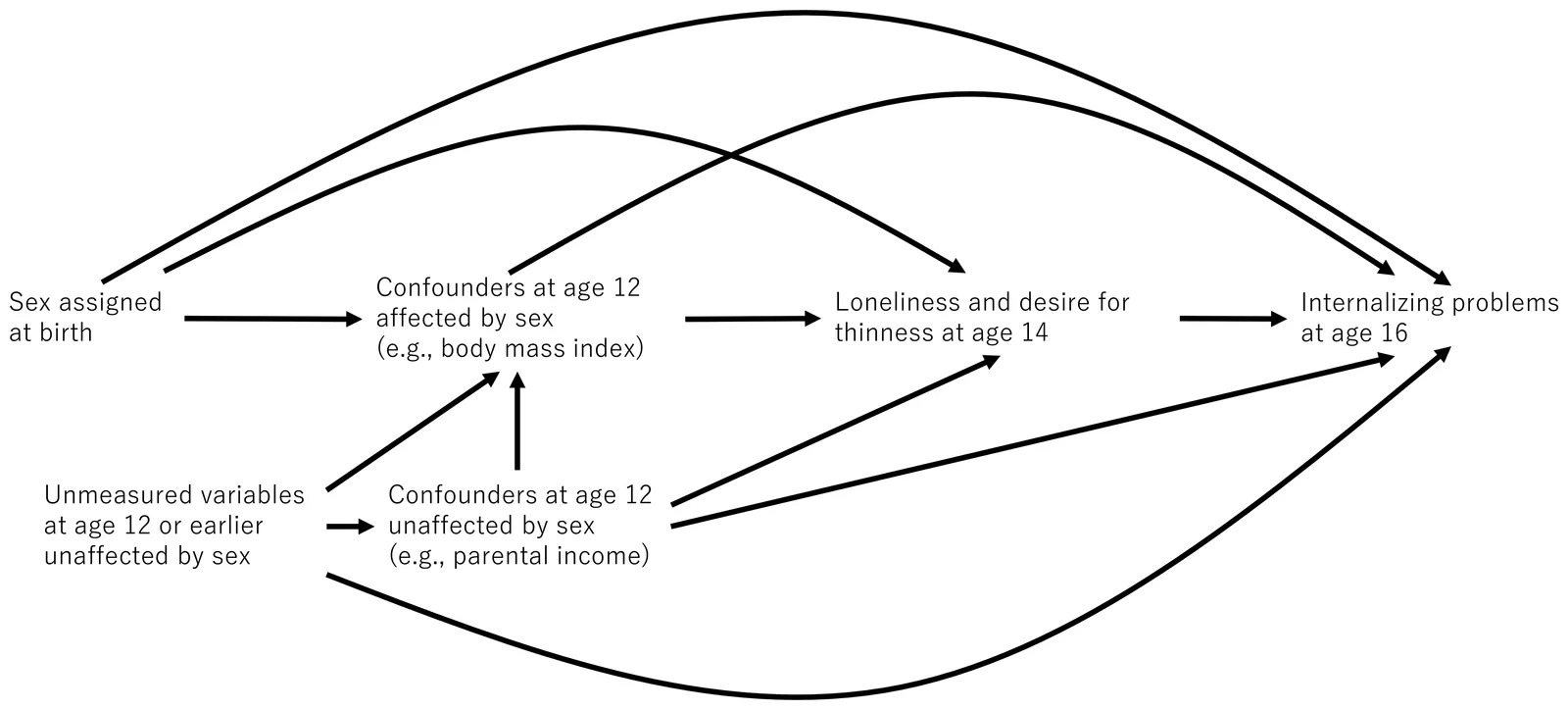
Directed acyclic graph showing hypothesized associations between female sex, confounders at age 12, mediators at age 14, and internalizing problems at age 16. The diagram shows sex assigned at birth, confounders measured at age 12, loneliness and desire for thinness measured at age 14, and internalizing problems measured at age 16. Confounders are classified according to whether they may be affected by sex, with unmeasured variables at age 12 or earlier also represented.

A series of workshops and meetings was held with young people from December 2022 to September 2025, using a structured sticky note wall method to elicit and cluster their perspectives. The involvement of young people began during the interpretation of previous analyses, when they contributed ideas and helped create an initial mindmap that informed subsequent study design and questions^6^. Through these workshops, young people and researchers co-identified three potential mechanisms: loneliness, desire for thinness, and gender-related stressors. However, because data on gender-related stressors were not available in the present dataset, our analyses focused on the first two. These constructs, which have also been associated with mental health problems in previous research^20–22^, anchored our analytic focus (Supplementary Table 1). Fig. 1 illustrates the co-production process, including (a) the Bridging Divides funding proposal development and preliminary discussions held both online and offline, (b) the workshops and meetings where young people proposed potential mediators, (c) researchers’ mapping of data availability and causal frameworks onto young people’s ideas, and (d) the follow-up workshop, where young people and researchers further discussed the selected mediators. Details are provided in Supplementary Method 1. In the present study, we used data from 3,171 adolescents in the Tokyo Teen Cohort (TTC). Fig. 2 presents a directed acyclic graph showing the hypothesized associations between female sex, confounders at age 12, mediators at age 14, and internalizing problems at age 16. The present study was conducted in partnership with the Bridging Divides Young Persons Advisory Group, who are listed as group authors, to ensure that young people’s perspectives were embedded throughout the study design.

## Results

### Co-production overview and the mechanisms informed by young people

Supplementary Table 1 summarizes the perspectives voiced by young people during the co-production sessions on why and how loneliness and desire for thinness may contribute to worse mental health in girls. For loneliness, they described potential contributing factors: criticism for standing out, which leads to feeling lonely; exclusion from group chats; the feeling that no one listens; having few emotionally safe and authentic friendships; continuous efforts to fit in with friends, otherwise feeling lonely; and the expectation to stay strong because their parents relied on them, which made it hard to share worries and only deepened their loneliness. For the desire for thinness, young people described the following factors: teasing about appearance; comparisons of body shape intensified by mobile phone use; the sense of being judged as potential romantic targets based on body shape; perceptions that school uniforms only look good if one is thin; and the belief that women must be thin to succeed.

### Baseline characteristics

Table 1 summarizes the baseline characteristics of the study population assessed at age 12. The analytic sample included 3,171 adolescents, of whom 1,487 were girls, and 1,684 were boys. The mean age was 12.2 years. Girls were less likely than boys to engage in physical activity once per month or more, to spend one hour or more per day playing games, and to report bullying victimization or physical punishment. Girls also had lower problematic internet use scores. Depressive symptoms, suicidal ideation, self-harm, and emotional symptoms were slightly more common among girls, whereas anxiety was slightly more common among boys. There were no substantial sex differences in other baseline characteristics, including the proportion of missing data.

### Internalizing problems at ages 12 and 16

Table 2 summarizes internalizing problems at ages 12 and 16. Results were synthesized from 20 multiple imputations by chained equations using Rubin’s rules. For depressive symptoms, emotional symptoms, and anxiety, prevalence increased among girls but decreased among boys from ages 12 to 16. For suicidal ideation, prevalence increased in both sexes, with a steeper rise among girls. For self-harm, prevalence decreased in both sexes, but the decline was smaller in girls than in boys. All of these patterns contributed to the substantially higher prevalence of internalizing problems among girls than boys at age 16.

### Potential mediators at age 14

Table 2 summarizes the mediators at age 14. Results were similarly synthesized from 20 multiple imputations. Girls had substantially higher prevalence of loneliness and desire for thinness compared with boys.

### Causal mediation analysis

**Fig. 3:**
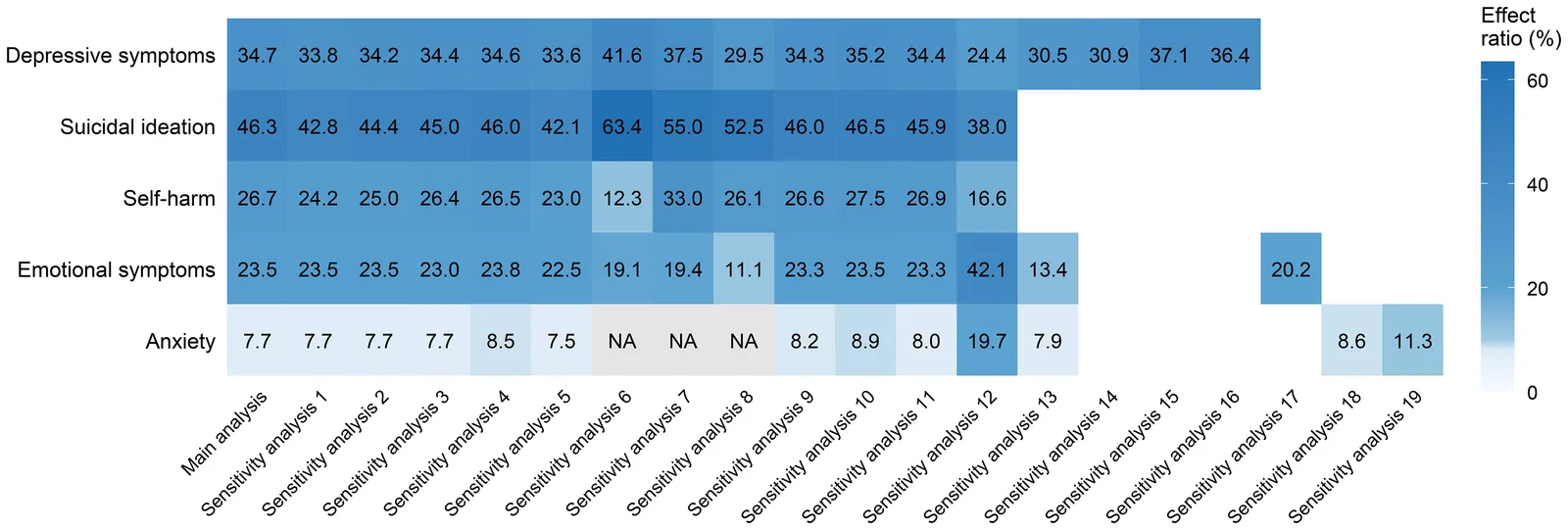
Effect ratio by loneliness and desire for thinness at age 14 in the association between female sex and internalizing problems at age 16. Sensitivity analysis 1: calculating the effect ratio as the interventional indirect effect divided by the total effect. Sensitivity analysis 2: calculating the effect ratio as the interventional indirect effect divided by the overall effect. Sensitivity analysis 3: adjusting for attention-deficit/hyperactivity. Sensitivity analysis 4: accounting for the interaction between bullying victimization and mediators. Sensitivity analysis 5: using a more restrictive definition of loneliness (“always” feeling lonely). Sensitivity analysis 6: using an alternative dichotomization of desire for thinness (including “I somewhat want to be thinner”). Sensitivity analysis 7: using body dissatisfaction measured using the Stunkard Figure Rating Scale instead of desire for thinness. Sensitivity analysis 8: including problematic internet use at age 14 as a mediator. Sensitivity analysis 9: assuming a joint distribution of mediators (desire for thinness conditional on loneliness). Sensitivity analysis 10: assuming a joint distribution of mediators (loneliness conditional on desire for thinness). Sensitivity analysis 11: using semiparametric generalized additive models to allow for flexible functional forms. Sensitivity analysis 12: using inverse probability weighting to account for censoring of mediators and outcomes, instead of multiple imputation. Sensitivity analysis 13: analyzing depressive symptoms, emotional symptoms, and anxiety symptoms as continuous variables. Sensitivity analysis 14: excluding the loneliness item from the Short Mood and Feelings Questionnaire to evaluate depressive symptoms. Sensitivity analysis 15: using an alternative dichotomization cutoff (≥ 7) after excluding the loneliness item from the Short Mood and Feelings Questionnaire to evaluate depressive symptoms. Sensitivity analysis 16: using an alternative dichotomization cutoff (≥ 8) after excluding the loneliness item from the Short Mood and Feelings Questionnaire to evaluate depressive symptoms. Sensitivity analysis 17: using an alternative dichotomization cutoff (≥ 4) on the Strengths and Difficulties Questionnaire emotional symptoms scale (including the borderline range) to evaluate emotional symptoms. Sensitivity analysis 18: using a different cutoff (T-score of 60) to evaluate anxiety. Sensitivity analysis 19: using a different cutoff (T-score of 70) to evaluate anxiety. NA, not applicable.

Fig. 3 and Table 3 summarize the results of the causal mediation analysis. Estimation of natural direct and indirect effects relies on the assumption that mediator-outcome confounding affected by sex does not exist^23^, which is unlikely to hold. To address this issue, we instead estimated a randomized interventional analogue of natural direct and indirect effects (referred to here as interventional direct and indirect effects)^23–28^. This approach, implemented using the parametric mediational g-formula with mediators randomly drawn from their counterfactual distributions, does not require such an assumption^24^. We examined mediation via both loneliness and desire for thinness rather than examining each mediator separately, as these mediators may interact with one another^29^. Details are shown in the Methods section. We adjusted for mediator-outcome confounders both unaffected by sex (i.e., age; parental income; living arrangements; neighborhood cohesion; IQ) and affected by sex (i.e., body mass index; physical activity; relationships with mother, father, and friends; bullying victimization; physical punishment; problematic internet use; time spent gaming; coffee consumption; pubertal development; belief in equal housework; and each internalizing problem). Note that prior internalizing problems are important mediator-outcome confounders affected by exposure, and failure to adjust for these variables results in confounding bias^13,14^. Importantly, when performed appropriately, the g-formula allows adjustment for confounders affected by exposure without introducing collider bias, representing a key advantage of this approach^30^. The analysis revealed evidence of mediation via both loneliness and desire for thinness at age 14 in the association between female sex and internalizing problems at age 16. The effect through these mediators relative to the total effect was 34.7% for depressive symptoms, 46.3% for suicidal ideation, 26.7% for self-harm, 23.5% for emotional symptoms, and 7.7% for anxiety. There was strong evidence supporting interventional indirect effects for depressive symptoms (risk difference (RD) 2.6%, 95% confidence interval (CI) 1.1%–4.2%) and suicidal ideation (RD 1.2%, 95% CI 0.2%–2.3%), while results for self-harm, emotional symptoms, and anxiety were less precise, as indicated by the wider CIs. As a negative control outcome analysis, we repeated the same interventional mediation analysis using asthma at age 16 as the outcome. We found no evidence of mediation (Supplementary Table 2). To contextualize the findings, we also analyzed each mediator individually, although this approach does not capture mediator-mediator interactions (Supplementary Table 3). This analysis likewise indicated evidence of mediation via each of loneliness and desire for thinness.

### Sensitivity analysis

To evaluate the robustness of the findings, we conducted 19 sensitivity analyses addressing alternative effect-ratio definitions, additional confounder adjustment, potential effect modification, mediator definitions, inclusion of an additional mediator, joint mediator distributions, flexible model specification, missing-data handling, and alternative outcome definitions (Fig. 3 and Supplementary Tables 4–22). Overall, the findings were broadly consistent with the main analysis. Full results are provided in Supplementary Results.

## Discussion

In this Japanese cohort, girls showed worse mental health at age 16, consistent with past reports. In early adolescence (age 12), sex differences in internalizing problems were small, with boys even showing slightly higher prevalence of anxiety. By late adolescence (age 16), however, girls showed consistently and substantially higher prevalence across all mental health outcomes. These shifts reflected increases in depressive symptoms, emotional symptoms, and anxiety among girls and decreases among boys, as well as a steeper rise in suicidal ideation and a smaller decline in self-harm among girls. This developmental pattern partly diverges from findings in an Australian study, where prevalence increased across adolescence in both sexes, with a steeper rise among girls^7^. The differences in the two mediators at age 14 mirrored young people’s perspectives, with a higher prevalence of loneliness and desire for thinness among girls. These mediators may reflect experiences that are particularly relevant to girls, such as appearance-based teasing, so their higher prevalence among girls may not be surprising. We interpreted these differences within an ecosocial framework, which conceptualizes gendered experiences as embodied expressions of social and structural contexts^31^.

We co-produced the present study with young people, in partnership with the Bridging Divides Young Persons Advisory Group. Their lived experiences informed the research questions and the selection of the two mediators at age 14, namely loneliness and desire for thinness. Positioning these factors informed by young people up front increased the interpretability of the findings in a real-world context. It also addresses the underrepresentation of adolescents in mental health research^15–19^ by grounding the analytic focus in lived experience rather than researcher assumptions and by centering modifiable pathways suggested by young people. As a result, the selected mediators have practical relevance, highlighting areas where intervention could directly address young people's perspectives, as subsequently discussed.

Using the parametric mediational g-formula, we found evidence of mediation via loneliness and desire for thinness at age 14 in the association between female sex and internalizing problems at age 16. The evidence was strong for interventional indirect effects on depressive symptoms and suicidal ideation; the effect through these factors reached up to almost half of the total effect of female sex on these outcomes. Note that this should be interpreted together with the magnitude of the interventional indirect effect; for example, the corresponding RD was 1.2% for suicidal ideation despite a relatively large effect ratio. These findings were robust across an extensive set of sensitivity analyses. On the other hand, parent-reported measures, particularly for anxiety, showed weaker evidence. This may be because parent-reported measures tend to underestimate negative outcomes in adolescents^32^. Both mediators analyzed in the present study could be modifiable pathways linking female sex to adolescent mental health and thus merit consideration as targets for prevention and policy. As various intervention strategies can target these mediators, the choice and implementation of such interventions warrant careful consideration.

Findings from the present study suggest that greater attention to structural and social contexts may be warranted in response to the higher prevalence of internalizing problems in girls. The higher prevalence of loneliness among girls may reflect gendered interpersonal expectations, as suggested by young people in the co-production process. These perspectives are consistent with a UNICEF technical note stating that girls are often expected to care for others and engage in close relationships, which may increase sensitivity to interpersonal stress^33^. Loneliness may be modifiable, and its remission is associated with lower subsequent risk of mental health problems^34^, supporting its consideration as a potential intervention target. A systematic review suggests that interventions such as social support groups and increasing social interaction may be beneficial for addressing loneliness^35^. Although this evidence is not specific to girls, tailoring support to address potential contributors to loneliness identified in the present co-production process, such as relational exclusion, may be particularly relevant. Policies that limit the dissemination of content that promotes a desire for thinness, including advertising and social media posts, could help reduce harmful appearance pressures. Specific strategies may include advertising restrictions, algorithm transparency, and public awareness campaigns. To ensure these reforms are effective and responsive to the needs of young people, educational and policy initiatives should include their meaningful participation.

The present study has several strengths. The temporal ordering of sex assigned at birth, confounders at age 12, mediators at age 14, and outcomes at age 16 reduced the risk of reverse causation. We accounted for sex-mediator and mediator-mediator interactions, the former of which represents one of the key considerations in mediation analysis^13^, and the latter of which captures how mediators interact with each other, an aspect that is overlooked when analyzing single mediators individually^14,29^. Furthermore, we computed interventional effects via the parametric mediational g-formula, which does not rely upon the assumption of no female sex-induced mediator-outcome confounding. This represents a key advantage over typical mediation analyses computing natural effects, which assume such confounding does not exist, an assumption that may not hold in practice. In addition, we conducted an extensive series of sensitivity analyses examining additional confounder adjustment, additional mediator inclusion, alternative model specifications, alternative mediator definitions, alternative assumptions about the joint distribution of the mediators, and alternative definitions of outcomes.

Several limitations should be acknowledged. First, despite multi-wave assessments and extensive adjustments, residual confounding cannot be excluded given the observational design^36,37^. Important confounders such as physical injuries^38,39^ and school climate^40^ were not included, and adverse childhood experiences^41,42^ were only partially captured by physical punishment. Second, some measures were not formally validated in Japanese, which may introduce measurement bias. In addition, mediators relied on single-item measures rather than validated multi-item psychometric instruments. Although we examined associations with theoretically related constructs within the cohort to support construct validity, measurement bias may still have influenced the mediation estimates. Future studies using validated multi-item scales would help better capture these mechanisms. Third, while the co-production process enhanced the clinical relevance of the study, this was conducted after data collection within this longitudinal cohort, meaning that the mechanisms suggested by young people were examined using existing rather than co-produced measures. Fourth, boys may be less likely than girls to disclose or recognize internalizing problems, which may have led to underestimation of their symptoms and overestimation of observed sex differences. Similarly, loneliness and desire for thinness were self-reported subjective experiences, and differential reporting by sex may have influenced the observed findings. The direction of this bias for the mediation estimates is difficult to determine, because it depends on the relative magnitude of measurement bias in the mediators and the outcomes. Fifth, although we used multiple imputation and conducted inverse-probability-of-missingness weighting as a complementary analysis, the possibility of missing not at random cannot be ruled out. Sixth, the mediators were assessed at the same time point without considering potential temporal ordering. While sensitivity analyses assuming the joint distribution of mediators, where loneliness affects desire for thinness and vice versa, suggested similar results, future studies should explicitly model the temporal ordering of these mediators. Finally, the cohort is from a socioeconomically advantaged, highly urbanized city (Tokyo), with a predominantly ethnic Japanese population in a country with conservative gender norms. While lower-income households were oversampled to improve socioeconomic representation, the sample may still not fully reflect the broader population. Therefore, the findings may not be generalizable to rural areas of Japan, more socioeconomically or ethnically diverse populations, or countries with different gender norms and media environments.

Notwithstanding the specific socio-cultural context of Japan in relation to gender, no country is immune from gendered framings, which are highly influential on adolescent mental health. Our findings suggest that social intermediates may contribute to the higher prevalence of internalizing problems in girls, highlighting potential targets for interventions and policies informed by young people.

## Methods

### Co-production

As described above, we adopted a co-production approach to incorporate young people’s perspectives into study design and interpretation (Fig. 1). Workshops and meetings with young people informed the selection of loneliness and desire for thinness as candidate mediating pathways; further details are provided in Supplementary Method 1.

### Participants

We used longitudinal data with multiple measurement waves from the TTC, a population-based cohort^43^. The inclusion criteria were: (1) birth between September 2002 and August 2004, (2) residence in Setagaya, Mitaka, or Chofu (Tokyo), and (3) written parental consent. A total of 3,171 participants were randomly selected using the basic resident registers of three municipalities. Data collection occurred at four time points when participants were age 10 (November 2012–January 2015), 12 (August 2014–January 2017, follow-up rate 94.8%), 14 (March 2017–March 2019, follow-up rate 84.1%), and 16 (February 2019–September 2021, follow-up rate 82.5%). Further details are provided in Supplementary Method 2. The present analyses used data from ages 12, 14, and 16. At each wave, trained interviewers administered self-report questionnaires. All 3,171 adolescents were included in the analyses using imputation methods, described in the statistical analysis section. The study was approved by the Institutional Review Boards of the Tokyo Metropolitan Institute of Medical Science (12-35), the University of Tokyo (10057), and SOKENDAI (2012002). Written informed consent was obtained from all parents.

### Sex

Information on sex (as assigned at birth) was obtained from the basic resident registers, which record sex as a binary category (female/male). Sex was recorded on the initial birth record by medical professionals based on genital phenotype.

### Internalizing problems

For depressive symptoms at ages 12 and 16, we administered the Short Mood and Feelings Questionnaire (SMFQ), a standard 13-item self-report measure^44,45^. Response options included “always” (2 points), “sometimes” (1 point), and “never” (0 points). Item scores were summed to yield a total score ranging from 0–26, with higher scores representing more severe symptoms. Depressive symptoms were dichotomized using a cut-off score of 8 on the SMFQ^44^. In sensitivity analyses, we also examined depressive symptoms as a continuous measure and after excluding the item assessing loneliness (which was also used to measure loneliness in this study), applying alternative cutoffs (≥ 7 and ≥ 8).

Suicidal ideation and self-harm were assessed at ages 12 and 16 using dichotomous variables derived from self-report questions. Suicidal ideation was measured with the item “Have you ever felt that life was not worth living?” with four response options: (1) “no,” (2) “rather no,” (3) “rather yes,” and (4) “yes.” Responses of 3 or 4 were coded as indicating suicidal ideation. For the causal mediation analysis, suicidal ideation at age 16 was instead assessed using a current-state item (“Do you currently feel that life is not worth living?”) to minimize the risk of reverse causation. The prevalence of current-state suicidal ideation at age 16 was 7.0% in girls (95% CI 5.7%–8.4%) and 4.3% in boys (95% CI 3.3%–5.3%). Among participants endorsing current-state suicidal ideation, 98.8% also endorsed lifetime suicidal ideation at the same wave (95% CI 95.6%–99.9%), supporting concurrent consistency. To provide additional support for construct validity, we examined associations with theoretically related measures at age 16. As expected, the current-state suicidal ideation item was associated with self-harm (odds ratio 11.28, 95% CI 7.36–17.22) and higher SMFQ depressive symptom scores (mean difference 8.02, 95% CI 7.25–8.79). Self-harm was assessed with the item “In the past year, have you ever intentionally harmed yourself?” At age 12, the response options were binary (yes/no). At age 16, five response options were used: (1) “never,” (2) “once,” (3) “2–5 times,” (4) “6–10 times,” and (5) “more than 10 times,” and responses of 2 or higher were coded as indicating self-harm.

To measure emotional symptoms, we used the parent-reported SDQ^46^ at ages 12 and 16. The emotional symptoms subscale consists of five items assessing worries, unhappiness, somatic complaints, fears, and nervousness. Response options included “not true” (0 points), “somewhat true” (1 point), and “certainly true” (2 points). Item scores were summed to yield a total score ranging from 0–10, with higher scores indicating more severe emotional symptoms. Emotional symptoms were dichotomized using a cut-off score corresponding to the abnormal range (≥ 5). In sensitivity analyses, we additionally examined an alternative dichotomization including the borderline range (≥ 4).

To measure anxiety, we used the parent-reported CBCL^47^ at ages 12 and 16. To reduce participant burden, we selected 86 items in total, including 14 items from the original 16-item CBCL anxiety scale^48^. Response categories were “not true,” “somewhat or sometimes true,” and “very true or often true.” Total scores ranged from 0–28, with higher values reflecting greater anxiety symptoms. Anxiety was dichotomized with a T-score of 65^49^. To provide additional support for construct validity, we examined its association with the SDQ emotional symptoms subscale^50^, which captures closely related emotional difficulties. As expected, the anxiety score was positively associated with SDQ emotional symptoms (*r* 0.70, 95% CI 0.67–0.71), supporting the construct validity of the measure in this cohort. In sensitivity analyses, we further examined anxiety as a continuous measure and using alternative cutoffs (T-scores of 60 and 70).

Although we evaluated both self-reported and parent-reported internalizing problems, we considered self-reported measures the primary indicators, as parent reports may underestimate adolescents’ internalizing difficulties^32^.

### Potential mediators

Potential mediators were assessed at age 14 and coded as binary variables. Variable construction was informed by a co-production process in which adolescents and researchers contributed to the operationalization and coding decisions (Fig. 1 and Supplementary Table 1).

Loneliness was measured using the item “I felt lonely” from the SMFQ^44,51^. Response options included “always,” “sometimes,” and “never.” The variable was dichotomized as “never” versus “sometimes or always.” To provide additional support for construct validity, we examined its association with the CBCL social withdrawal subscale, which captures closely related behaviors of social withdrawal and isolation. As expected, loneliness was associated with social withdrawal (mean difference 0.73, 95% CI 0.53–0.92). In addition, prior analyses from the same cohort have shown that longitudinal patterns of loneliness across adolescence were associated with subsequent mental health problems, including psychotic experiences, depressive symptoms, anxiety symptoms, and diminished well-being, with persistent loneliness showing a dose-response relationship with later outcomes^34^. Together, these findings support the construct validity of this measure in this population. In a sensitivity analysis, we also used a more restrictive definition of loneliness, coding the variable as present only when participants reported “always” feeling lonely.

Desire for thinness was assessed with the item, “Do you want to be thinner than you are now?” Response options were: (1) “I want to be thinner,” (2) “I somewhat want to be thinner,” (3) “I somewhat do not want to be thinner,” and (4) “I do not want to be thinner.” Endorsement of option 1 was coded as positive for the desire for thinness, while all other responses were coded as negative. We note that this item was designed to capture endorsement of the desire for thinness, rather than general body satisfaction. To provide additional support for construct validity, we examined its associations with related body-image and eating-related indicators available in the dataset. As expected, desire for thinness was associated with body dissatisfaction measured using the Stunkard Figure Rating Scale^52^ (mean difference 1.78, 95% CI 1.67–1.89), as well as with binge eating (odds ratio 2.54, 95% CI 2.03–3.16) and purging (odds ratio 9.85, 95% CI 4.20–27.23). These associations were in the expected direction and support the construct validity of this measure of desire for thinness. In sensitivity analyses, we applied an alternative dichotomization including participants who responded “I somewhat want to be thinner” and also examined body dissatisfaction as an alternative mediator. Note that we did not use body dissatisfaction in the main analysis because it may capture body-image concerns beyond desire for thinness, including a desire to be larger.

Although data availability limited us to single-item measures rather than validated multi-item psychometric instruments, we made substantial efforts to support validity. These included the use of a co-production process with young people to inform the selection of the variables and enhance their practical relevance, empirical evaluation of associations with theoretically related constructs within the cohort, and consideration of prior longitudinal evidence from the same population. Taken together, these steps provide supportive evidence that the selected items were reasonable indicators of the intended constructs in the present study.

### Covariates

We adjusted for potential confounders measured at age 12, guided by clinical knowledge. Covariate selection was determined *a priori* based on the assumed causal structure (Fig. 2) and substantive knowledge regarding potential confounding, rather than on observed baseline imbalance. No variable would confound the associations between female sex and internalizing problems (i.e., *sex-outcome confounding*) or the associations of female sex with loneliness and desire for thinness (i.e., *sex-mediator confounding*)^53^. Nevertheless, there may be variables that confound the associations of loneliness and desire for thinness with internalizing problems (i.e., *mediator-outcome confounding*). Confounders unaffected by sex included age; parental income^54^; living arrangements^55^; neighborhood cohesion^56,57^; and IQ^58^. Confounders affected by sex included body mass index^59^; physical activity^60^; relationships with mother, father, and friends^61^; bullying victimization^62^; physical punishment^63^; problematic internet use^64^; time spent gaming^65^; coffee consumption^66^; pubertal development^67^; and belief in equal housework^68^. For each analysis, we further included internalizing problems assessed at age 12. Details on covariates are provided in Supplementary Method 3.

### Statistical analysis

Analyses were conducted using R version 4.5.1. We first examined the prevalence of internalizing problems at ages 12 and 16. Point estimates and variances of prevalence were calculated separately for girls and boys, with prevalence defined as a simple proportion. Corresponding 95% CIs were also calculated for each group.

Next, we examined the prevalence of potential mediators. Point estimates and variances of prevalence were calculated separately for girls and boys, with prevalence defined as a simple proportion. Corresponding 95% CIs were then obtained for each group.

Finally, we performed causal mediation analysis. The assumptions underlying the analysis are described in Supplementary Method 4. We applied the parametric mediational g-formula^24^ to estimate interventional direct and indirect effects of female sex on adolescent internalizing problems via loneliness and desire for thinness. These mediators were treated as distinct variables rather than a single composite mediator. As mentioned in the results section, we examined mediation via both mediators, as they may interact with one another^29^. We adjusted for confounders measured at age 12, including both those unaffected by sex (i.e., age; parental income; living arrangements; neighborhood cohesion; IQ) and affected by sex (i.e., body mass index; physical activity; relationships with mother, father, and friends; bullying victimization; physical punishment; problematic internet use; time spent gaming; coffee consumption; pubertal development; belief in equal housework; and each internalizing problem). Details of the parametric mediational g-formula are summarized in Supplementary Method 5. We assessed the fit of the logistic models used for outcomes by comparing observed versus predicted probabilities across 10 quantile-based groups based on predicted probability, which showed no major discrepancies (Supplementary Fig. 1). To assess covariate overlap, we examined baseline distributions across groups defined by sex and loneliness or desire for thinness at age 14, which indicated sufficient overlap across groups (Supplementary Tables 23 and 24). We further conducted a positivity assessment by evaluating the estimated probabilities^69^, which showed no indication of positivity violations across imputed datasets (Supplementary Table 25).

We handled missing data via multiple imputation by chained equations using random forest algorithms and combined estimates across 20 imputed datasets. To examine attrition patterns, we summarized retention from age 12 to age 16 stratified by sex and baseline internalizing symptomatology, defined as the presence of at least one internalizing problem at age 12 (Supplementary Table 26). For the prevalence analysis, means and CIs were computed using Rubin’s rules to combine results across imputations^70^. For causal mediation analysis, parameter estimates were obtained using the MI Boot approach with pooled samples^71^, which combined the 20 imputed datasets with 200 bootstrap resamples for each dataset (resulting in 4,000 total replicates^72,73^). Point estimates were computed as the mean across all replicates, and 95% CIs were determined using the percentile method. Monte Carlo error for the MI Boot point estimates was less than 0.01 percentage points for all estimands within each outcome. As described above, we conducted 19 sensitivity analyses to assess the robustness of the mediation results; full specifications are provided in Supplementary Method 6. The mediation analyses were reported in accordance with the AGReMA statement^74^.

## Supplementary Material

Supplementary Information

## Figures and Tables

**Table 1:** Baseline characteristics of the study population assessed at age 12

Variables	Overall (*N* = 3,171)	Girls (*N* = 1,487)	Boys (*N* = 1,684)	SMD
Age (years), mean (SD)	12.2 (0.3)	12.2 (0.3)	12.2 (0.3)	0.04
Missing, n (%)	4 (0.1)	2 (0.1)	2 (0.1)	
Parental income, JPY, n (%)				0.10
< 5,000,000	448 (14.1)	189 (12.7)	259 (15.4)	
5,000,000 to 9,999,999	1,300 (41.0)	634 (42.6)	666 (39.5)	
≥ 10,000,000	681 (21.5)	321 (21.6)	360 (21.4)	
Missing	742 (23.4)	343 (23.1)	399 (23.7)	
Living arrangements, n (%)				0.01
With father	2,730 (92.7)	1,288 (92.8)	1,442 (92.7)	
Separated from father	214 (7.3)	100 (7.2)	114 (7.3)	
Missing	227 (7.2)	99 (6.7)	128 (7.6)	
Neighborhood cohesion, mean (SD)	12.9 (2.89)	12.9 (2.89)	12.9 (2.88)	0.01
Missing, n (%)	177 (5.6)	88 (5.9)	89 (5.3)	
IQ, mean (SD)	110 (14.9)	111 (14.5)	109 (15.2)	0.15
Missing, n (%)	631 (19.9)	295 (19.8)	336 (20.0)	
Body mass index, mean (SD)	17.9 (2.51)	17.9 (2.43)	17.9 (2.58)	0.00
Missing, n (%)	617 (19.5)	287 (19.3)	330 (19.6)	
Physical activity, n (%)				0.22
Once per month or more	2,462 (77.6)	1,110 (74.6)	1,352 (80.3)	
Less than once per month	387 (12.2)	235 (15.8)	152 (9.0)	
Missing	322 (10.2)	142 (9.5)	180 (10.7)	
Relationship with mother, mean (SD)	3.79 (0.986)	3.79 (0.987)	3.80 (0.986)	0.02
Missing, n (%)	406 (12.8)	192 (12.9)	214 (12.7)	
Relationship with father, mean (SD)	3.43 (1.19)	3.45 (1.18)	3.42 (1.21)	0.02
Missing, n (%)	406 (12.8)	192 (12.9)	214 (12.7)	
Relationships with friends, mean (SD)	3.52 (0.922)	3.56 (0.929)	3.48 (0.915)	0.08
Missing, n (%)	406 (12.8)	192 (12.9)	214 (12.7)	
Bullying victimization, n (%)				0.21
No	2,522 (79.5)	1,245 (83.7)	1,277 (75.8)	
Yes or somewhat yes	478 (15.1)	169 (11.4)	309 (18.3)	
Missing	171 (5.4)	73 (4.9)	98 (5.8)	
Physical punishment, n (%)				0.14
Never or rarely	1,840 (58.0)	901 (60.6)	939 (55.8)	
Sometimes, often, or always	906 (28.6)	379 (25.5)	527 (31.3)	
Missing	425 (13.4)	207 (13.9)	218 (12.9)	
Problematic internet use, mean (SD)	3.94 (4.16)	3.33 (3.74)	4.49 (4.44)	0.28
Missing, n (%)	202 (6.4)	84 (5.6)	118 (7.0)	
Time spent gaming, n (%)				0.56
Less than one hour per day	2,125 (67.0)	1,186 (79.8)	939 (55.8)	
One hour per day or longer	873 (27.5)	229 (15.4)	644 (38.2)	
Missing	173 (5.5)	72 (4.8)	101 (6.0)	
Coffee consumption, n (%)				0.03
None	2,680 (84.5)	1,265 (85.1)	1,415 (84.0)	
One cup per day or more	245 (7.7)	110 (7.4)	135 (8.0)	
Missing	246 (7.8)	112 (7.5)	134 (8.0)	
Pubertal development, n (%)				0.00
Tanner stage 1	381 (12.0)	194 (13.0)	187 (11.1)	
Tanner stages 2–5	2,066 (65.2)	1,053 (70.8)	1,013 (60.2)	
Missing	724 (22.8)	240 (16.1)	484 (28.7)	
Belief in equal housework, n (%)				0.03
Strongly agree or agree	1,825 (57.6)	871 (58.6)	954 (56.7)	
Disagree or strongly disagree	1,040 (32.8)	482 (32.4)	558 (33.1)	
Missing	306 (9.7)	134 (9.0)	172 (10.2)	
Depressive symptoms, n (%)				0.03
No	2,035 (64.2)	949 (63.8)	1,086 (64.5)	
Yes	444 (14.0)	218 (14.7)	226 (13.4)	
Missing	692 (21.8)	320 (21.5)	372 (22.1)	
Suicidal ideation, n (%)				0.06
No	1,988 (62.7)	924 (62.1)	1,064 (63.2)	
Yes	518 (16.3)	259 (17.4)	259 (15.4)	
Missing	665 (21.0)	304 (20.4)	361 (21.4)	
Self-harm, n (%)				0.06
No	2,202 (69.4)	1,011 (68.0)	1,191 (70.7)	
Yes	278 (8.8)	141 (9.5)	137 (8.1)	
Missing	691 (21.8)	335 (22.5)	356 (21.1)	
Emotional symptoms, n (%)				0.05
No	2819 (88.9)	1321 (88.8)	1498 (89.0)	
Yes	169 (5.3)	88 (5.9)	81 (4.8)	
Missing	183 (5.8)	78 (5.2)	105 (6.2)	
Anxiety, n (%)				0.04
No	2,462 (77.6)	1,159 (77.9)	1,303 (77.4)	
Yes	263 (8.3)	114 (7.7)	149 (8.8)	
Missing	446 (14.1)	214 (14.4)	232 (13.8)	

SD, standard deviation; SMD, standardized mean difference

**Table 2:** Prevalence of internalizing problems and mediators.

Measure	Variable	Report type	Age	Prevalence	
				Girls % (95% CI)	Boys % (95% CI)
Outcome	Depressive symptoms	Self-report	12 16	15.6 (13.7–17.5) 15.9 (13.9–18.0)	14.3 (12.6–16.1) 8.2 (6.7–9.7)
Outcome	Suicidal ideation	Self-report	12 16	19.2 (17.1–21.4) 29.8 (27.2–32.4)	17.4 (15.4–19.4) 18.1 (15.9–20.4)
Outcome	Self-harm	Self-report	12 16	10.8 (9.1–12.5) 5.6 (4.4–6.8)	9.4 (7.9–10.8) 2.3 (1.6–3.1)
Outcome	Emotional symptoms	Parent-report	12 16	5.9 (4.7–7.1) 6.3 (5.0–7.5)	4.9 (3.8–5.9) 3.1 (2.2–3.9)
Outcome	Anxiety	Parent-report	12 16	8.0 (6.6–9.4) 9.1 (7.6–10.7)	9.1 (7.7–10.5) 5.8 (4.7–7.0)
Mediator	Loneliness	Self-report	14	13.2 (11.4–15.1)	6.0 (4.8–7.3)
Mediator	Desire for thinness	Self-report	14	41.4 (38.3–44.5)	11.1 (9.2–12.9)

CI, confidence interval. Prevalence was obtained using Rubin’s rules.

**Table 3:** Mediation via both loneliness and desire for thinness at age 14 in the association between female sex and internalizing problems at age 16.

	Self-report						Parent-report		
Mediators and estimates	Depressive symptoms		Suicidal ideation		Self-harm		Emotional symptoms		Anxiety
Loneliness and desire for thinness									
Total effect RD, % [95% CI]	7.7 [5.3, 10.1]		2.8 [1.1, 4.6]		3.3 [1.8, 4.8]		3.4 [1.8, 5.1]		3.5 [1.6, 5.4]
Overall effect RD, % [95% CI]	7.6 [5.2, 10.0]		2.7 [1.0, 4.5]		3.2 [1.7, 4.7]		3.4 [1.8, 5.1]		3.5 [1.6, 5.4]
Interventional direct effect RD, % [95% CI]	5.0 [2.4, 7.6]		1.5 [-0.2, 3.4]		2.4 [0.7, 4.1]		2.6 [0.9, 4.4]		3.2 [1.1, 5.4]
Interventional indirect effect RD, % [95% CI]	2.6 [1.1, 4.2]		1.2 [0.2, 2.3]		0.8 [-0.1, 1.7]		0.8 [-0.2, 1.9]		0.3 [-0.9, 1.4]
Difference RD, % [95% CI]	0.1 [-0.4, 0.5]		0.1 [-0.3, 0.5]		0.1 [-0.2, 0.3]		0.0 [-0.4, 0.4]		0.0 [-0.5, 0.5]
Effect ratio, %	34.7		46.3		26.7		23.5		7.7

RD, risk difference, CI, confidence interval Suicidal ideation at age 16 was assessed using a current-state item to minimize the risk of reverse causation, which explains the discrepancy between the total effect in this table and the sex difference in Table 2. Missing data were handled using multiple imputation by chained equations. Parametric mediational g-formula was used for mediation analysis. The analysis accounted for sex-mediator and mediator-mediator interactions. Overall effect was computed as interventional direct effect + interventional indirect effect; difference was computed as total effect – overall effect; effect ratio was computed as (interventional indirect effect + difference) / total effect. To address mediator-outcome confounding, we adjusted for confounders, including both those unaffected by sex (i.e., age; parental income; living arrangements; neighborhood cohesion; and IQ) and those affected by sex (i.e., body mass index; physical activity; relationships with mother, father, and friends; bullying victimization; physical punishment; problematic internet use; time spent gaming; coffee consumption; pubertal development; belief in equal housework; and each internalizing problem). Estimates were obtained using MI Boot with pooled samples, based on 20 imputed datasets and 200 bootstrap samples per dataset (4,000 replicates in total); point estimates were calculated as the mean of replicates, and 95% confidence intervals were derived using the percentiles.

## Data Availability

The data analyzed in this study are not publicly available due to usage restrictions and licensing agreements with the Tokyo Teen Cohort. Certain variables are proprietary and cannot be shared openly. De-identified data are available to researchers upon request to the co-corresponding authors, subject to approval by the Tokyo Teen Cohort steering committee. Requests will normally be reviewed within four weeks. Access is subject to the Tokyo Teen Cohort data-use conditions, and approved researchers may be required to enter into a data-use agreement. Requests should be directed to Zui C. Narita (zuinarita@ncnp.go.jp) or Syudo Yamasaki (yamasaki-sd@igakuken.or.jp).

## Code availability

Analyses were conducted using R version 4.5.1. The parametric mediational g-formula was implemented using custom code. The code supporting the main analyses is available on GitHub (https://github.com/ZuiCNarita/Loneliness-thinness-sex-differences-mediation).
