# Supplementary material for: Loneliness and desire for thinness explain sex differences in adolescent internalizing problems
Source: Nat Hum Behav. Author manuscript; Available in PMC 2026 Oct 11. (PMC7619547; doi:10.1038/s41562-026-02578-2)
Supplement: Supplementary file 1 [file EMS218036-supplement-Supplementary_Information.pdf]

Supplementary Results | Sensitivity analyses

To evaluate the robustness of the findings, we conducted 19 sensitivity analyses (Fig. 3 and Supplementary Tables 4–22). Sensitivity Analyses 1 and 2 calculated alternative effect ratios excluding the difference term. Specifically, we calculated the ratio of the interventional indirect effect divided by the total effect and the ratio of the interventional indirect effect divided by the overall effect. The results were comparable to the main analysis (Fig. 3 and Supplementary Tables 4 and 5). Sensitivity Analysis 3 included attention-deficit/hyperactivity as an additional confounder. Although this variable itself has psychopathological aspects, it could also serve as a confounder in relation to internalizing problems according to the modified disjunctive cause criterion1; the results were comparable to the main analysis (Fig. 3 and Supplementary Table 6). Sensitivity Analysis 4 accounted for the interaction between bullying victimization and the mediators and showed similar results to the main analysis (Fig. 3 and Supplementary Table 7). Sensitivity Analysis 5 used a more restrictive definition of loneliness, defining the mediator as present only when participants reported “always” feeling lonely rather than “sometimes or always.” The results were comparable to the main analysis, and the overall pattern of mediation remained consistent (Fig. 3 and Supplementary Table 8). Sensitivity Analysis 6 used an alternative dichotomization of desire for thinness, defining the mediator as present when participants reported either “I want to be thinner” or “I somewhat want to be thinner,” rather than only “I want to be thinner.” The overall pattern of mediation was similar to the main analysis, while the effect ratio for self-harm was somewhat diminished and that for anxiety was not estimable (Fig. 3 and Supplementary Table 9). Sensitivity Analysis 7 used body dissatisfaction measured using the Stunkard Figure Rating Scale2 instead of desire for thinness; higher effect ratios were observed for depressive symptoms, suicidal ideation, and self-harm, while the effect ratio for anxiety was not estimable (Fig. 3 and Supplementary Table 10). Sensitivity Analysis 8 included problematic internet use at age 14 as a mediator. This analysis included the interaction of problematic internet use with female sex, loneliness, and desire for thinness. The results were generally similar to the main analysis, while the effect ratio for emotional symptoms was somewhat diminished and that for anxiety was not estimable (Fig. 3 and Supplementary Table 11). Sensitivity Analysis 9 specified a joint distribution of the mediators by fitting the model for desire for thinness conditional on loneliness. The findings remained similar to the main analysis (Fig. 3 and Supplementary Table 12). Sensitivity Analysis 10 specified a joint distribution of the mediators by fitting the model for loneliness conditional on desire for thinness, with similarly consistent results (Fig. 3 and Supplementary Table 13). Sensitivity Analysis 11 used semiparametric generalized additive models to allow for flexible functional forms. The results were comparable to the main analysis (Fig. 3 and Supplementary Table 14). Sensitivity Analysis 12 used inverse probability weighting instead of multiple imputation. Although the estimates fluctuated slightly, evidence for mediation was retained (Fig. 3 and Supplementary Table 15). Sensitivity Analysis 13 analyzed depressive symptoms, emotional symptoms, and anxiety symptoms as continuous variables and showed a largely similar pattern of mediation to the main analysis, while the effect ratio for emotional symptoms was somewhat diminished (Fig. 3 and Supplementary Table 16). Sensitivity Analysis 14 excluded the loneliness item from the Short Mood and Feelings Questionnaire when evaluating depressive symptoms and showed similar evidence of mediation (Fig. 3 and Supplementary Table 17). Sensitivity Analyses 15 and 16 re-estimated depressive symptoms after excluding the loneliness item from the Short Mood and Feelings Questionnaire3,4 and then applied two alternative cutoffs (≥7 and ≥8), with consistent evidence of mediation across both analyses (Fig. 3 and Supplementary Tables 18 and 19). Sensitivity Analysis 17 used an alternative dichotomization of emotional symptoms based on the Strengths and Difficulties

Questionnaire5, including the borderline range (≥4), and yielded results comparable to the main analysis (Fig. 3 and Supplementary Table 20). Sensitivity Analyses 18 and 19 used alternative cutoffs for anxiety based on the Child Behavior Checklist anxiety scale6 (T-scores of 60 and 70 instead of 65), and the results were generally unchanged (Fig. 3 and Supplementary Tables 21 and 22).

References

1. VanderWeele, T. J. Principles of confounder selection. Eur. J. Epidemiol. 34, 211–219 (2019).
2. Stunkard, A. J., Sørensen, T. & Schulsinger, F. Use of the Danish Adoption Register for the study of obesity and thinness. Res. Publ. Assoc. Res. Nerv. Ment. Dis. 60, 115–120 (1983).
3. Angold, A., Costello, E. J., Messer, S. C. & Pickles, A. Development of a short questionnaire for use in epidemiological studies of depression in children and adolescents. Int. J. Methods Psychiatr. Res. 5, 237–249 (1995).
4. Sharp, C., Goodyer, I. M. & Croudace, T. J. The Short Mood and Feelings Questionnaire (SMFQ): a unidimensional item response theory and categorical data factor analysis of self-report ratings from a community sample of 7-through 11-year-old children. J. Abnorm. Child Psychol. 34, 379–391 (2006).
5. Goodman, R. The Strengths and Difficulties Questionnaire: a research note. J. Child Psychol. Psychiatry 38, 581–586 (1997).
6. Iijima, Y. et al. Response inhibition and anxiety in adolescents: Results from a population-based community sample. J. Affect. Disord. 246, 89–95 (2019).

Supplementary Method 1 | Details of co-production process.

We adopted a co-production approach to incorporate young people's perspectives into the study design and interpretation (Fig. 1). The co-production team consisted of participants of the TTC born between 2002 and 2004 (now at ages 21 to 23). Through regular newsletters sent to the TTC participants, we invited volunteers to join a co-production team; 11 joined the team. Of these, seven currently attend monthly team meetings and constitute the core group. The current mean age of members is 22 years, and many have firsthand experience of mental health difficulties during adolescence and challenges with help-seeking; their lived experiences provided practically relevant insights for research and practice.

The involvement of young people began in December 2022, when previous analyses on sex differences in adolescent mental health were being interpreted. At that stage, the young people contributed ideas, and based on their input, an initial mindmap was drawn, which informed the subsequent study design and research questions. Multiple workshops were then conducted with young people up to April 2025. These workshops followed a sticky note wall approach, where young people wrote their ideas on sticky notes and placed them on shared boards, enabling collective discussion and the clustering of themes. Young people shared their views on possible explanations for the higher prevalence of internalizing problems in girls, e.g., why sex differences in mental health may emerge in early adolescence.

In April 2025, structured discussion sessions were held to bring together young people and researchers. During these meetings, the young people examined multiple sticky note walls to organize and synthesize the overall discussion. At the same time, researchers discussed the appropriate methods to decipher the pathways from female sex to internalizing problems.

Researchers then reviewed the suggestions from the young people and evaluated the feasibility for quantitative analysis, taking into account both data availability in the TTC and the study's causal framework. Based on this process, a set of potential mediators was selected.

In September 2025, the young people and researchers met again to further discuss and refine these potential mediators and initial findings. Discussions were again organized using a sticky note wall approach that allowed participants to post and cluster ideas visually as a way to explore why and how these potential mediators might be important in the context of the higher prevalence of internalizing problems in girls.

The formal analysis and manuscript writing were subsequently conducted based on this series of discussions. This process ensured that the study addressed questions of direct relevance to young people while maintaining methodological rigor. Because the workshops took place after data collection, the mechanisms suggested by the young people were examined using existing rather than co-developed measures.

Supplementary Method 2 | Sampling and recruitment process for the Tokyo Teen Cohort.

The Tokyo Teen Cohort was established based on the Tokyo Early Adolescence Survey (T-EAS). The sampling frame comprised all children listed in the resident registries of three municipalities in metropolitan Tokyo (Setagaya, Mitaka, and Chofu) who were born between September 2002 and August 2004 (N = 18,830). Due to budgetary constraints, 14,553 children were randomly selected from this population and invited to participate in T-EAS. Of these, 10,234 were successfully contacted, and 4,478 agreed to participate at age 10.

For the establishment of the TTC cohort, the research team aimed to recruit approximately 3,000 participants for longitudinal follow-up. To ensure sufficient representation of lower-income households, all 620 T-EAS participants with an annual household income below 5,000,000 JPY were invited to participate. In addition, 2,551 participants were randomly selected from the remaining 3,858 T-EAS participants. In total, 3,171 children were invited to join the TTC, forming the baseline sample for the present study (see figure below).

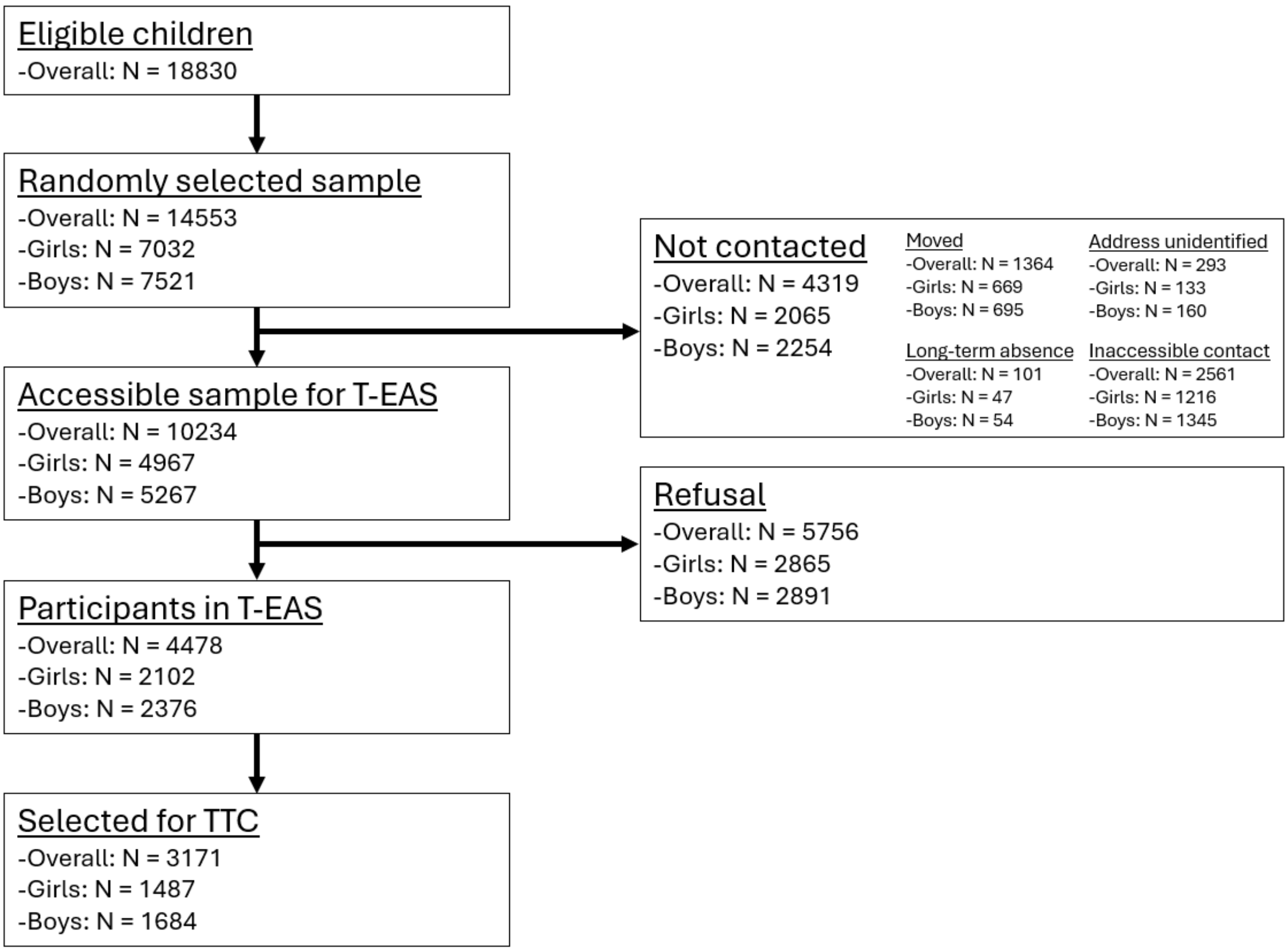

Supplementary Method 3 | Details on the measurement of covariates at age 12.

Mediator-outcome confounders unaffected by sex included age (months, continuous); parental income (Japanese Yen < 5,000,000, 5,000,000 to 9,999,999, or ≥ 10,000,000, categorical); living arrangements (dichotomous, with father vs. separated from father); neighborhood cohesion (continuous); and IQ (continuous). Mediator-outcome confounders affected by sex included body mass index (continuous); physical activity (dichotomous, once per month or more vs. less than once per month); relationships with mother, father, and friends (continuous); bullying victimization (dichotomous, no vs. yes or somewhat yes); physical punishment (dichotomous, never or rarely vs. sometimes, often, or always); problematic internet use (continuous); time spent gaming (dichotomous, less than one hour per day vs. one hour per day or longer); coffee consumption (dichotomous, none vs. one cup per day or more); pubertal development (dichotomous, Tanner stage 1 vs. stages 2–5); and belief in equal housework (dichotomous, strongly agree or agree vs. disagree or strongly disagree). For each analysis, we further included internalizing problems (dichotomous).

Neighborhood cohesion was assessed using the Neighborhood Collective Efficacy scale[1], where a higher total score indicates greater neighborhood cohesion (Cronbach's α, 0.87). IQ was evaluated using the short form of the Wechsler Intelligence Scale for Children-Third Edition[2]. The relationships with mother, father, and friends were evaluated using the Network of Relationships Inventory[3], where a higher score indicates better relationships. Problematic internet use was evaluated using the modified version of the Compulsive Internet Use Scale[4], where a higher total score indicates greater problematic internet use (Cronbach's α, 0.87). Pubertal development was assessed using Tanner staging based on breast development among girls and genital development among boys. Tanner stage was dichotomized as stage 1 versus stages 2–5. Belief in equal housework was assessed using a single item asking whether men and women should do housework equally.

Problematic internet use was also discussed as a potential mechanism during the co-production workshops. However, based on prior empirical evidence and substantive knowledge regarding sex differences[5], we specified an *a priori* causal framework in which problematic internet use was treated as a mediator-outcome confounder rather than a candidate mediator. Previous analyses in the same cohort and related literature have consistently shown that problematic internet use is more prevalent among boys than girls, making a pathway from female sex to problematic internet use unlikely. Accordingly, problematic internet use was treated as a potential confounder in the main mediation analyses, although we conducted additional sensitivity analyses in which problematic internet use was examined as a potential mediator.

References

1. Nakanishi M, Yamasaki S, Ando S, et al. Neighborhood Social Cohesion and Dementia-Related Stigma Among Mothers of Adolescents in the Pre- and Current COVID-19 Period: An Observational Study Using Population-Based Cohort Data. J Alzheimers Dis. 2022;88(2):493-502.
2. Inada N, Kamio Y. Short forms of the Japanese version WISC-3 for assessment of children with autism spectrum disorders. Jpn J Child Adolesc Psychiatr (Japanese). 2010;51:11-19.
3. Furman W, Buhrmester D. The Network of Relationships Inventory: Behavioral Systems Version. *Int J Behav Dev*. 2009;33(5):470-478.
4. Meerkerk GJ, Van Den Eijnden RJJM, Vermulst AA, Garretsen HFL. The Compulsive Internet Use Scale (CIUS): some psychometric properties. Cyberpsychol Behav. 2009;12(1):1-6.

5. Narita Z, Ando S, Yamasaki S, Miyashita M, DeVylder J, Yamaguchi S, Hosozawa M, Nakanishi M, Hiraiwa-Hasegawa M, Furukawa TA, Kasai K, Nishida A. Association of Problematic Internet Use with Psychotic Experiences and Depression in Adolescents: A Cohort Study. Schizophr Bull. 2024;51(1):198-207.

Supplementary Method 4 | Assumptions underlying the mediation analyses.

For mediation analysis, we did not analyze natural effects but their randomized interventional analogues[1–6]. This is because female sex would affect variables that may confound mediator-outcome associations, violating the cross-world counterfactual independence assumption required for identifying natural effects. Analyses of randomized interventional analogues do not rely upon this assumption and can be identified under three other assumptions: (i) no sex-outcome confounding, (ii) no sex-mediator confounding, and (iii) no unmeasured mediator-outcome confounding. The first two assumptions are likely to hold because sex is randomly assigned at birth. To address the third assumption, we adjusted for a rich set of confounders measured at age 12. The parametric mediational g-formula relies on the correct specification of the statistical models used in the simulation procedure. Binary variables were modeled using logistic regression and continuous variables using linear regression. The simulation step assumed Bernoulli distributions for binary variables and normal distributions for continuous variables.

References

1. VanderWeele, T. J. & Tchetgen Tchetgen, E. J. Mediation analysis with time varying exposures and mediators. J. R. Stat. Soc. Series B Stat. Methodol. 79, 917–938 (2017).
2. Yamamuro, S., Shinozaki, T., Iimuro, S. & Matsuyama, Y. Mediational g-formula for time-varying treatment and repeated-measured multiple mediators: Application to atorvastatin's effect on cardiovascular disease via cholesterol lowering and anti-inflammatory actions in elderly type 2 diabetics. Stat. Methods Med. Res. 30, 1782–1799 (2021).
3. Miles, C. H. On the causal interpretation of randomised interventional indirect effects. J. R. Stat. Soc. Series B Stat. Methodol. 85, 1154–1172 (2023).
4. Nguyen, T. Q., Schmid, I. & Stuart, E. A. Clarifying causal mediation analysis for the applied researcher: Defining effects based on what we want to learn. Psychol. Methods 26, 255–271 (2021).
5. Vansteelandt, S. & Daniel, R. M. Interventional effects for mediation analysis with multiple mediators. Epidemiology 28, 258–265 (2017).
6. Rudolph, K. E., Williams, N. T. & Diaz, I. Practical causal mediation analysis: extending nonparametric estimators to accommodate multiple mediators and multiple intermediate confounders. Biostatistics 25, 997–1014 (2024).

Supplementary Method 5 | Details of the parametric mediational g-formula.

The analysis proceeded in several steps. First, we specified models for the following variables: 1) confounders at age 12 affected by sex conditional on sex and confounders unaffected by sex at age 12; 2) each mediator at age 14 conditional on sex and the full confounder set at age 12; and 3) internalizing problems at age 16 conditional on sex, the full confounder set at age 12, and two mediators at age 14, including sex-mediator and mediator-mediator interactions. Then, we sequentially performed a random draw of the values for sex-affected confounders and mediators from the fitted models in the previous steps by substituting possible levels for sex (i.e., boy or girl) and the drawn levels for these variables, which estimated the counterfactual distributions under the hypothetical intervention on sex. Here, we assumed a marginal distribution of the mediators under the hypothetical intervention on sex, while multiple sensitivity analyses were performed assuming a joint distribution instead. Finally, we simulated counterfactual values of internalizing problems under different counterfactual values of sex and mediators. Risk differences were computed as contrasts between standardized mean predicted counterfactual risks from the logistic outcome models, rather than as coefficients from linear probability models. This allowed us to obtain (i) the total effect (TE) estimated under the standard g-formula, (ii) the overall effect (OE) (sum of interventional direct and indirect effects), (iii) the interventional direct effect (IDE), (iv) the interventional indirect effects (IIE) through the two mediators, and (v) the difference between the TE and OE.

The difference term represents the discrepancy between the original TE and the part of the effect recovered by the interventional decomposition. In this framework, TE compares the original outcome contrast, allowing downstream variables of exposure, including mediators and exposure-induced mediator-outcome confounders, to follow their natural joint process under each exposure level. By contrast, IDE and IIE are defined by stochastic interventions guided by mediator-specific marginal counterfactual distributions. By "marginal," we mean the counterfactual distribution of each mediator conditional on baseline covariates, but not the joint counterfactual distribution of all mediators and not distributions conditional on other mediators or intermediate mediator-outcome confounders under exposure intervention. Therefore, OE obtained by summing the IDE and IIE does not necessarily reproduce the actual TE. The resulting difference may reflect aspects of the natural dependence structure between the counterfactual variables, including mediator-mediator dependence and cross-world mediator-outcome dependence induced by intermediate mediator-outcome confounders. If this difference is large, the decomposition of the TE using interventional effects is less informative; we therefore evaluated whether the difference was sufficiently small.

To quantify the interventional effect decomposition, we calculated the proportion not attributable to the IDE: (IIE + difference)/TE, which equals (TE – IDE)/TE. We included the difference term in the numerator of the main effect-ratio calculation because we wished to define the ratio relative to the original TE, as the part of the TE not captured by IDE, i.e., the TE minus the IDE. This quantity should not be interpreted as a pure mediator-specific proportion of mediated effect, but rather as the proportion of the original TE not attributable to the IDE. We also calculated the effect ratio without the difference term (i.e., IIE/TE) and the proportion of the IIE among the OE (i.e., IIE/OE) as Sensitivity Analyses 1 and 2. Because the main analysis assumed interventions on mediators based on marginal counterfactual distributions, the mediator-mediator dependence term discussed by Vansteelandt and Daniel[1] was not separately decomposed as a named component; joint-distribution specifications were examined in Sensitivity Analyses 9 and 10.

Estimands (i)–(v) were defined on the RD scale. Model specification and parameter definitions used in the mediation analysis are described below.

For each mediator at age 14 (dichotomous), we fitted the following model:
logit{*P*(Mediator at age 14| Sex assigned at birth, Confounders at age 12 unaffected by sex, Confounders at age 12 affected by sex)}
= b_0 + b_1 Sex assigned at birth
+ b_2 Confounders at age 12 unaffected by sex + b_3 Confounders at age 12 affected by sex

For each internalizing problem at age 16 (dichotomous), we fitted the following model:
logit{*P*(Internalizing problem at age 16| Sex assigned at birth, Confounders at age 12 unaffected by sex, Confounders at age 12 affected by sex, Loneliness at age 14, Desire for thinness at age 14)}
= c_0 + c_1 Sex assigned at birth + c_2 Loneliness at age 14
+ c_3 Desire for thinness at age 14
+ c_4 Sex assigned at birth * Loneliness at age 14
+ c_5 Sex assigned at birth * Desire for thinness at age 14
+ c_6 Loneliness at age 14 * Desire for thinness at age 14
+ c_7 Confounders at age 12 unaffected by sex + c_8 Confounders at age 12 affected by sex

TE = $E[Y_1 M1_1 M2_1] - E[Y_0 M1_0 M2_0]$
OE = $E[Y_1 G1_1 G2_1] - E[Y_0 G1_0 G2_0]$
IDE = $E[Y_1 G1_0 G2_0] - E[Y_0 G1_0 G2_0]$
IIE = $E[Y_1 G1_1 G2_1] - E[Y_1 G1_0 G2_0]$
Difference = TE – OE
where Y is the outcome, M is the mediator, and G is the randomly permuted mediator.

Reference

1. Vansteelandt, S. & Daniel, R. M. Interventional effects for mediation analysis with multiple mediators. Epidemiology 28, 258–265 (2017).

Supplementary Method 6 | Details of sensitivity analyses.

Sensitivity analysis 1: Calculation of the effect ratio as the interventional indirect effect divided by the total effect.
Sensitivity analysis 2: Calculation of the effect ratio as the interventional indirect effect divided by the overall effect.
Sensitivity analysis 3: Adjustment for attention-deficit/hyperactivity, which could act as a mediator–outcome confounder under the modified disjunctive cause criterion.
Sensitivity analysis 4: Inclusion of an interaction term between bullying victimization and the mediators, to examine potential effect modification.
Sensitivity analysis 5: A more restrictive definition of loneliness (mediator defined as "always" feeling lonely), to assess sensitivity to mediator definition.
Sensitivity analysis 6: An alternative dichotomization of desire for thinness including participants who responded "I somewhat want to be thinner," to assess sensitivity to mediator definition.
Sensitivity analysis 7: An alternative specification of the mediator using body dissatisfaction measured using the Stunkard Figure Rating Scale instead of desire for thinness, to assess sensitivity to mediator definition.
Sensitivity analysis 8: Inclusion of problematic internet use at age 14 as an additional mediator, although prior evidence suggested it was unlikely to represent a meaningful pathway.
Because the primary analyses simulated mediators from their marginal distributions, we additionally examined specifications assuming a joint distribution of the mediators.
Sensitivity analysis 9: Desire for thinness modeled conditional on loneliness.
Sensitivity analysis 10: Loneliness modeled conditional on desire for thinness.
Sensitivity analysis 11: Use of semiparametric generalized additive models to allow for flexible functional forms.
Sensitivity analysis 12: Use of inverse probability weighting to account for censoring of mediators and outcomes, instead of multiple imputation.
Sensitivity analysis 13: Depressive symptoms, emotional symptoms, and anxiety symptoms analyzed as continuous outcomes rather than dichotomous variables, to examine sensitivity to outcome specification.
Sensitivity analysis 14: Depressive symptoms defined using the Short Mood and Feelings Questionnaire score excluding the loneliness item, to address potential overlap between mediator and outcome measurement.
Because excluding the loneliness item yields a 12-item Short Mood and Feelings Questionnaire score, and rescaling the original cutoff of 8 on the 13-item scale corresponds to 7.4 on the 12-item scale ($8 \times 12/13$), we examined two alternative cutoffs.
Sensitivity analysis 15: Lower cutoff (≥ 7).
Sensitivity analysis 16: Higher cutoff (≥ 8).
For emotional symptoms, we examined an alternative dichotomization threshold.
Sensitivity analysis 17: Lower cutoff (the Strengths and Difficulties Questionnaire emotional symptoms score ≥ 4, i.e., including the borderline range as cases).
For anxiety, we examined alternative dichotomization thresholds.
Sensitivity analysis 18: Lower cutoff (T-score ≥ 60).
Sensitivity analysis 19: Higher cutoff (T-score ≥ 70).

Supplementary Figure 1 | Observed versus predicted probabilities for outcomes. For each outcome, participants were grouped into 10 quantile-based bins according to predicted probability, pooled across imputed datasets. Points show the observed event rate and mean predicted probability within each bin.

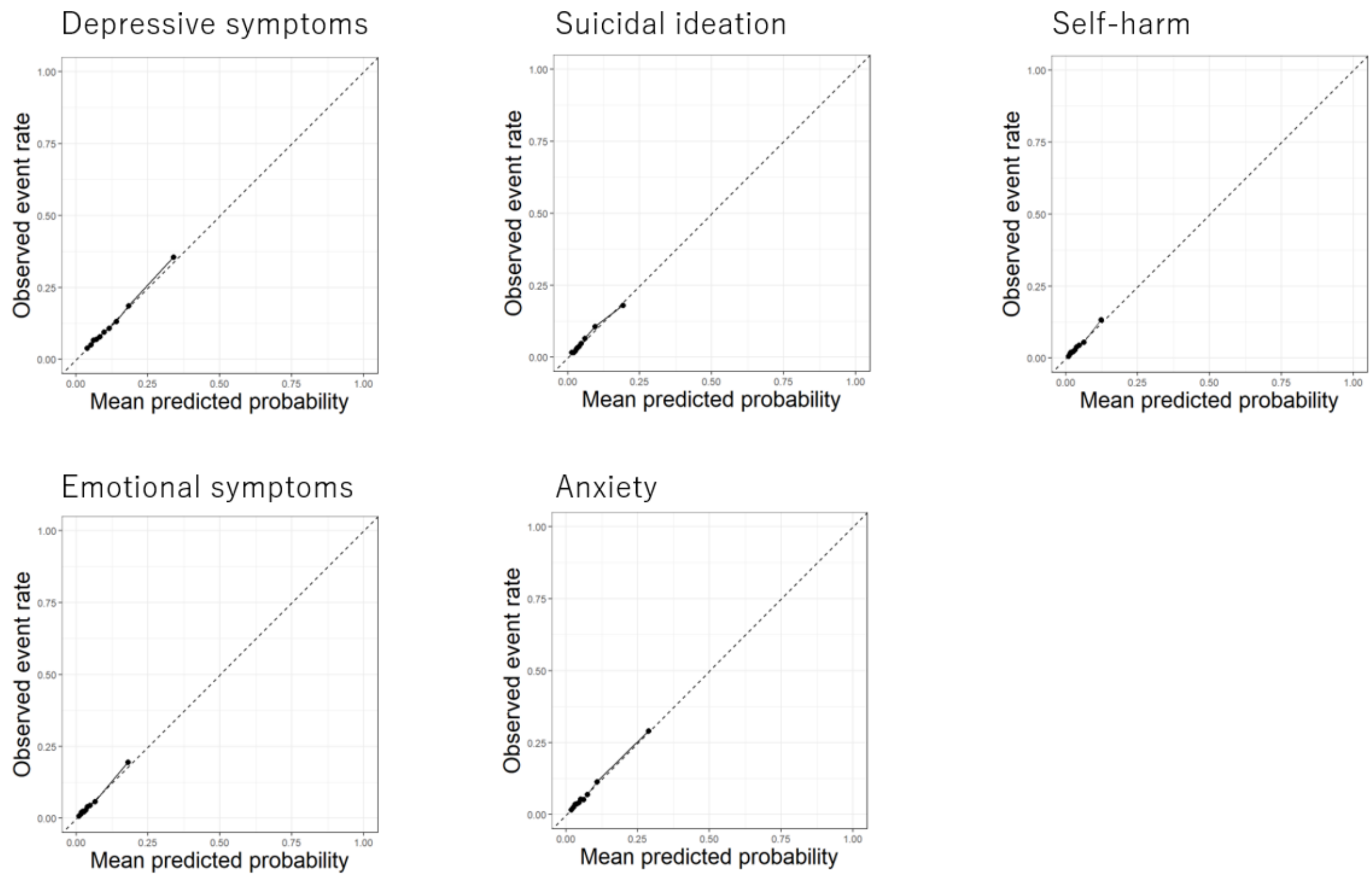

Supplementary Table 1 | Young people's perspectives from co-production discussions on potential mediators contributing to the higher prevalence of internalizing problems in girls.

Potential mediator	Young people's perspectives on why and how mediators may contribute to the higher prevalence of internalizing problems in girls
Loneliness	● Being criticized for standing out or taking initiative, often resulting in feeling lonely<br>● Feeling lonely when not included in social media group chats or conversations<br>● Perception that "no one listens to me," reinforcing a sense of loneliness<br>● Few friends with whom they can feel emotionally safe and authentic, leading to loneliness<br>● Continuous effort to fit in with friends, otherwise feeling lonely<br>● Because girls are more often relied upon by parents, they feel they must "be responsible," which makes it harder to share worries and leads to loneliness<br>● In contexts where being in a group is seen as the default, girls feel loneliness more readily when alone
Desire for thinness	● Being teased by boys about appearance<br>● Increased comparison of body shape after starting to use mobile phones<br>● Constant sense of being judged as a potential romantic target based on body shape<br>● Belief that school uniforms do not look good unless one is thin<br>● Comments and gazes on body shape from peers, sometimes even without malicious intent<br>● Perception that there are very few successful women who are not thin (e.g., mainly comedians, whereas men can succeed as athletes such as baseball or rugby players)

Supplementary Table 2 | Negative control outcome analysis examining asthma instead of mental health outcomes.

Mediators and estimates	Asthma
Loneliness and desire for thinness	
Total effect<br>RD, % [95% CI]	-2.0<br>[-4.7, 0.5]
Overall effect<br>RD, % [95% CI]	-2.0<br>[-4.8, 0.3]
Interventional direct effect<br>RD, % [95% CI]	-2.4<br>[-4.5, -0.2]
Interventional indirect effect<br>RD, % [95% CI]	0.4<br>[-0.7, 1.7]
Difference<br>RD, % [95% CI]	0.0<br>[-1.4, 1.4]
Effect ratio, %	NA[a]

RD, risk difference, CI, confidence interval
Missing data were handled using multiple imputation by chained equations.
Parametric mediational g-formula was used for mediation analysis.
The analysis accounted for sex-mediator and mediator-mediator interactions.
Overall effect was computed as interventional direct effect + interventional indirect effect; difference was computed as total effect – overall effect; effect ratio was computed as (interventional indirect effect + difference) / total effect.
To address mediator-outcome confounding, we adjusted for confounders, including both those unaffected by sex (i.e., age; parental income; living arrangements; neighborhood cohesion; and IQ) and those affected by sex (i.e., body mass index; physical activity; relationships with mother, father, and friends; bullying victimization; physical punishment; problematic internet use; time spent gaming; coffee consumption; pubertal development; belief in equal housework; and asthma).
Estimates were obtained using MI Boot with pooled samples, based on 20 imputed datasets and 200 bootstrap samples per dataset (4,000 replicates in total); point estimates were calculated as the mean of replicates, and 95% confidence intervals were derived using the percentiles.
[a] Not estimable because the interventional direct effect and the sum of the interventional indirect effect and the difference were in opposite directions.

Supplementary Table 3 | Analyzing each mediator individually.

Mediators and estimates	Self-report			Parent-report	
	Depressive symptoms	Suicidal ideation	Self-harm	Emotional symptoms	Anxiety
Loneliness					
Total effect<br>RD, % [95% CI]	7.8<br>[5.3, 10.2]	2.9<br>[1.2, 4.7]	3.3<br>[1.8, 4.8]	3.4<br>[1.8, 5.0]	3.5<br>[1.6, 5.5]
Overall effect<br>RD, % [95% CI]	7.7<br>[5.3, 10.1]	2.9<br>[1.1, 4.6]	3.3<br>[1.8, 4.8]	3.4<br>[1.8, 5.0]	3.5<br>[1.5, 5.5]
Interventional direct effect<br>RD, % [95% CI]	6.3<br>[3.9, 8.7]	2.3<br>[0.7, 4.0]	2.8<br>[1.3, 4.2]	3.2<br>[1.6, 4.9]	3.0<br>[1.1, 5.0]
Interventional indirect effect<br>RD, % [95% CI]	1.4<br>[0.7, 2.1]	0.5<br>[0.1, 1.0]	0.5<br>[0.2, 0.9]	0.2<br>[-0.2, 0.6]	0.5<br>[-0.1, 1.0]
Difference<br>RD, % [95% CI]	0.1<br>[-0.3, 0.5]	0.1<br>[-0.3, 0.4]	0.0<br>[-0.2, 0.2]	0.0<br>[-0.4, 0.4]	0.0<br>[-0.4, 0.5]
Effect ratio, %	18.4	21.7	16.6	6.0	14.4
Desire for thinness					
Total effect<br>RD, % [95% CI]	7.8<br>[5.4, 10.4]	2.9<br>[1.2, 4.8]	3.3<br>[1.8, 4.9]	3.5<br>[1.8, 5.1]	3.6<br>[1.7, 5.4]
Overall effect<br>RD, % [95% CI]	7.8<br>[5.5, 10.4]	3.0<br>[1.3, 4.7]	3.3<br>[1.8, 4.9]	3.5<br>[1.8, 5.2]	3.6<br>[1.7, 5.4]
Interventional direct effect<br>RD, % [95% CI]	6.3<br>[3.9, 9.0]	2.4<br>[0.7, 4.3]	2.8<br>[1.2, 4.6]	2.8<br>[1.1, 4.6]	3.1<br>[1.1, 5.2]
Interventional indirect effect<br>RD, % [95% CI]	1.5<br>[0.3, 2.8]	0.6<br>[-0.3, 1.5]	0.5<br>[-0.3, 1.3]	0.7<br>[-0.2, 1.6]	0.4<br>[-0.5, 1.4]
Difference<br>RD, % [95% CI]	0.0<br>[-0.4, 0.4]	-0.0<br>[-0.4, 0.4]	0.0<br>[-0.2, 0.2]	-0.0<br>[-0.4, 0.4]	0.0<br>[-0.5, 0.5]
Effect ratio, %	19.4	19.1	15.1	19.9	12.1

RD, risk difference, CI, confidence interval
Suicidal ideation at age 16 was assessed using a current-state item to minimize the risk of reverse causation, which explains the discrepancy between the total effect in this table and the sex difference in Table 2.
Missing data were handled using multiple imputation by chained equations.
Parametric mediational g-formula was used for mediation analysis.
The analysis accounted for sex-mediator interaction.
Overall effect was computed as interventional direct effect + interventional indirect effect; difference was computed as total effect – overall effect; effect ratio was computed as (interventional indirect effect + difference) / total effect.
To address mediator-outcome confounding, we adjusted for confounders, including both those unaffected by sex (i.e., age; parental income; living arrangements; neighborhood cohesion; and IQ) and those affected by sex (i.e., body mass index; physical activity; relationships with mother, father, and friends; bullying victimization; physical punishment; problematic internet use; time spent gaming; coffee consumption; pubertal development; belief in equal housework; and each internalizing problem).
Estimates were obtained using MI Boot with pooled samples, based on 20 imputed datasets and 200 bootstrap samples per dataset (4,000 replicates in total); point estimates were calculated as the mean of replicates, and 95% confidence intervals were derived using the percentiles.

Supplementary Table 4 | Sensitivity analysis 1: calculating the effect ratio as the interventional indirect effect divided by the total effect.

Mediators and estimates	Self-report			Parent-report	
	Depressive symptoms	Suicidal ideation	Self-harm	Emotional symptoms	Anxiety
Loneliness and desire for thinness					
Effect ratio, %	33.8	42.8	24.2	23.5	7.7

Suicidal ideation at age 16 was assessed using a current-state item to minimize the risk of reverse causation, which explains the discrepancy between the total effect in this table and the sex difference in Table 2.
Missing data were handled using multiple imputation by chained equations.
Parametric mediational g-formula was used for mediation analysis.
The analysis accounted for sex-mediator and mediator-mediator interactions.
Overall effect was computed as interventional direct effect + interventional indirect effect; difference was computed as total effect – overall effect.
To address mediator-outcome confounding, we adjusted for confounders, including both those unaffected by sex (i.e., age; parental income; living arrangements; neighborhood cohesion; and IQ) and those affected by sex (i.e., body mass index; physical activity; relationships with mother, father, and friends; bullying victimization; physical punishment; problematic internet use; time spent gaming; coffee consumption; pubertal development; belief in equal housework; and each internalizing problem).
Estimates were obtained using MI Boot with pooled samples, based on 20 imputed datasets and 200 bootstrap samples per dataset (4,000 replicates in total); point estimates were calculated as the mean of replicates, and 95% confidence intervals were derived using the percentiles.

Supplementary Table 5 | Sensitivity analysis 2: calculating the effect ratio as the interventional indirect effect divided by the overall effect.

Mediators and estimates	Self-report			Parent-report	
	Depressive symptoms	Suicidal ideation	Self-harm	Emotional symptoms	Anxiety
Loneliness and desire for thinness					
Effect ratio, %	34.2	44.4	25.0	23.5	7.7

Suicidal ideation at age 16 was assessed using a current-state item to minimize the risk of reverse causation, which explains the discrepancy between the total effect in this table and the sex difference in Table 2.
Missing data were handled using multiple imputation by chained equations.
Parametric mediational g-formula was used for mediation analysis.
The analysis accounted for sex-mediator and mediator-mediator interactions.
Overall effect was computed as interventional direct effect + interventional indirect effect; difference was computed as total effect – overall effect.
To address mediator-outcome confounding, we adjusted for confounders, including both those unaffected by sex (i.e., age; parental income; living arrangements; neighborhood cohesion; and IQ) and those affected by sex (i.e., body mass index; physical activity; relationships with mother, father, and friends; bullying victimization; physical punishment; problematic internet use; time spent gaming; coffee consumption; pubertal development; belief in equal housework; and each internalizing problem).
Estimates were obtained using MI Boot with pooled samples, based on 20 imputed datasets and 200 bootstrap samples per dataset (4,000 replicates in total); point estimates were calculated as the mean of replicates, and 95% confidence intervals were derived using the percentiles.

Supplementary Table 6 | Sensitivity analysis 3: adjusting for attention-deficit/hyperactivity.

Mediators and estimates	Self-report			Parent-report	
	Depressive symptoms	Suicidal ideation	Self-harm	Emotional symptoms	Anxiety
Loneliness and desire for thinness					
Total effect<br>RD, % [95% CI]	7.7<br>[5.3, 10.2]	2.9<br>[1.2, 4.6]	3.3<br>[1.8, 4.8]	3.4<br>[1.8, 5.1]	3.5<br>[1.6, 5.5]
Overall effect<br>RD, % [95% CI]	7.6<br>[5.2, 10.2]	2.8<br>[1.1, 4.5]	3.2<br>[1.7, 4.7]	3.4<br>[1.8, 5.1]	3.5<br>[1.6, 5.4]
Interventional direct effect<br>RD, % [95% CI]	5.1<br>[2.5, 7.7]	1.6<br>[-0.2, 3.5]	2.4<br>[0.8, 4.1]	2.6<br>[1.0, 4.4]	3.2<br>[1.1, 5.5]
Interventional indirect effect<br>RD, % [95% CI]	2.6<br>[1.1, 4.1]	1.2<br>[0.1, 2.3]	0.8<br>[-0.1, 1.7]	0.8<br>[-0.2, 1.9]	0.3<br>[-0.9, 1.4]
Difference<br>RD, % [95% CI]	0.1<br>[-0.4, 0.5]	0.1<br>[-0.3, 0.5]	0.1<br>[-0.2, 0.3]	-0.0<br>[-0.4, 0.4]	0.0<br>[-0.5, 0.5]
Effect ratio, %	34.4	45.0	26.4	23.0	7.7

RD, risk difference, CI, confidence interval
Suicidal ideation at age 16 was assessed using a current-state item to minimize the risk of reverse causation, which explains the discrepancy between the total effect in this table and the sex difference in Table 2.
Missing data were handled using multiple imputation by chained equations.
Parametric mediational g-formula was used for mediation analysis.
The analysis accounted for sex-mediator and mediator-mediator interactions.
Overall effect was computed as interventional direct effect + interventional indirect effect; difference was computed as total effect – overall effect; effect ratio was computed as (interventional indirect effect + difference) / total effect.
To address mediator-outcome confounding, we adjusted for confounders, including both those unaffected by sex (i.e., age; parental income; living arrangements; neighborhood cohesion; and IQ) and those affected by sex (i.e., body mass index; physical activity; relationships with mother, father, and friends; bullying victimization; physical punishment; problematic internet use; time spent gaming; coffee consumption; pubertal development; belief in equal housework; attention-deficit/hyperactivity; and each internalizing problem).
Estimates were obtained using MI Boot with pooled samples, based on 20 imputed datasets and 200 bootstrap samples per dataset (4,000 replicates in total); point estimates were calculated as the mean of replicates, and 95% confidence intervals were derived using the percentiles.

Supplementary Table 7 | Sensitivity analysis 4: accounting for the interaction between bullying victimization and mediators.

Mediators and estimates	Self-report			Parent-report	
	Depressive symptoms	Suicidal ideation	Self-harm	Emotional symptoms	Anxiety
Loneliness and desire for thinness					
Total effect<br>RD, % [95% CI]	7.7<br>[5.2, 10.3]	2.9<br>[1.2, 4.6]	3.3<br>[1.8, 4.8]	3.5<br>[1.9, 5.1]	3.5<br>[1.6, 5.4]
Overall effect<br>RD, % [95% CI]	7.6<br>[5.1, 10.2]	2.8<br>[1.1, 4.5]	3.2<br>[1.7, 4.8]	3.5<br>[1.9, 5.1]	3.5<br>[1.6, 5.4]
Interventional direct effect<br>RD, % [95% CI]	5.0<br>[2.5, 7.7]	1.6<br>[-0.2, 3.4]	2.4<br>[0.8, 4.1]	2.6<br>[0.9, 4.4]	3.2<br>[1.0, 5.4]
Interventional indirect effect<br>RD, % [95% CI]	2.6<br>[1.1, 4.1]	1.2<br>[0.2, 2.3]	0.8<br>[-0.1, 1.8]	0.8<br>[-0.2, 1.9]	0.3<br>[-0.9, 1.5]
Difference<br>RD, % [95% CI]	0.1<br>[-0.4, 0.5]	0.1<br>[-0.3, 0.5]	0.1<br>[-0.2, 0.3]	-0.0<br>[-0.4, 0.4]	-0.0<br>[-0.5, 0.5]
Effect ratio, %	34.6	46.0	26.5	23.8	8.5

RD, risk difference, CI, confidence interval
Suicidal ideation at age 16 was assessed using a current-state item to minimize the risk of reverse causation, which explains the discrepancy between the total effect in this table and the sex difference in Table 2.
Missing data were handled using multiple imputation by chained equations.
Parametric mediational g-formula was used for mediation analysis.
The analysis accounted for sex-mediator, mediator-mediator, and mediator-bullying victimization interactions.
Overall effect was computed as interventional direct effect + interventional indirect effect; difference was computed as total effect – overall effect; effect ratio was computed as (interventional indirect effect + difference) / total effect.
To address mediator-outcome confounding, we adjusted for confounders, including both those unaffected by sex (i.e., age; parental income; living arrangements; neighborhood cohesion; and IQ) and those affected by sex (i.e., body mass index; physical activity; relationships with mother, father, and friends; bullying victimization; physical punishment; problematic internet use; time spent gaming; coffee consumption; pubertal development; belief in equal housework; and each internalizing problem).
Estimates were obtained using MI Boot with pooled samples, based on 20 imputed datasets and 200 bootstrap samples per dataset (4,000 replicates in total); point estimates were calculated as the mean of replicates, and 95% confidence intervals were derived using the percentiles.

Supplementary Table 8 | Sensitivity analysis 5: using a more restrictive definition of loneliness ("always" feeling lonely).

Mediators and estimates	Self-report			Parent-report	
	Depressive symptoms	Suicidal ideation	Self-harm	Emotional symptoms	Anxiety
Loneliness and desire for thinness					
Total effect<br>RD, % [95% CI]	7.8<br>[5.3, 10.2]	3.0<br>[1.2, 4.7]	3.3<br>[1.8, 4.8]	3.5<br>[1.9, 5.2]	3.6<br>[1.6, 5.6]
Overall effect<br>RD, % [95% CI]	7.7<br>[5.3, 10.3]	3.0<br>[1.3, 4.7]	3.2<br>[1.7, 4.7]	3.5<br>[1.9, 5.2]	3.6<br>[1.7, 5.6]
Interventional direct effect<br>RD, % [95% CI]	5.2<br>[2.7, 7.9]	1.7<br>[-0.1, 3.6]	2.5<br>[0.9, 4.3]	2.7<br>[1.0, 4.5]	3.4<br>[1.2, 5.6]
Interventional indirect effect<br>RD, % [95% CI]	2.6<br>[1.1, 4.1]	1.2<br>[0.2, 2.4]	0.7<br>[-0.3, 1.6]	0.8<br>[-0.2, 1.9]	0.3<br>[-0.8, 1.4]
Difference<br>RD, % [95% CI]	0.1<br>[-0.4, 0.5]	0.0<br>[-0.4, 0.4]	0.1<br>[-0.2, 0.4]	-0.0<br>[-0.4, 0.4]	-0.0<br>[-0.5, 0.5]
Effect ratio, %	33.6	42.1	23.0	22.5	7.5

RD, risk difference, CI, confidence interval
Suicidal ideation at age 16 was assessed using a current-state item to minimize the risk of reverse causation, which explains the discrepancy between the total effect in this table and the sex difference in Table 2.
Missing data were handled using multiple imputation by chained equations.
Parametric mediational g-formula was used for mediation analysis.
The analysis accounted for sex-mediator and mediator-mediator interactions.
Overall effect was computed as interventional direct effect + interventional indirect effect; difference was computed as total effect – overall effect; effect ratio was computed as (interventional indirect effect + difference) / total effect.
To address mediator-outcome confounding, we adjusted for confounders, including both those unaffected by sex (i.e., age; parental income; living arrangements; neighborhood cohesion; and IQ) and those affected by sex (i.e., body mass index; physical activity; relationships with mother, father, and friends; bullying victimization; physical punishment; problematic internet use; time spent gaming; coffee consumption; pubertal development; belief in equal housework; and each internalizing problem).
Estimates were obtained using MI Boot with pooled samples, based on 20 imputed datasets and 200 bootstrap samples per dataset (4,000 replicates in total); point estimates were calculated as the mean of replicates, and 95% confidence intervals were derived using the percentiles.

Supplementary Table 9 | Sensitivity analysis 6: using an alternative dichotomization of desire for thinness (including "I somewhat want to be thinner").

Mediators and estimates	Self-report			Parent-report	
	Depressive symptoms	Suicidal ideation	Self-harm	Emotional symptoms	Anxiety
Loneliness and desire for thinness					
Total effect<br>RD, % [95% CI]	7.7<br>[5.3, 10.2]	2.9<br>[1.1, 4.6]	3.3<br>[1.7, 4.8]	3.4<br>[1.9, 5.1]	3.5<br>[1.5, 5.4]
Overall effect<br>RD, % [95% CI]	7.7<br>[5.3, 10.2]	2.9<br>[1.1, 4.5]	3.2<br>[1.7, 4.8]	3.4<br>[1.9, 5.1]	3.5<br>[1.6, 5.4]
Interventional direct effect<br>RD, % [95% CI]	4.5<br>[1.8, 7.4]	1.1<br>[-0.7, 3.0]	2.9<br>[0.8, 5.1]	2.8<br>[0.9, 4.8]	4.1<br>[1.3, 7.0]
Interventional indirect effect<br>RD, % [95% CI]	3.2<br>[1.1, 5.1]	1.8<br>[0.4, 3.1]	0.4<br>[-1.1, 1.7]	0.7<br>[-0.8, 1.9]	-0.6<br>[-2.6, 1.3]
Difference<br>RD, % [95% CI]	0.0<br>[-0.4, 0.4]	0.0<br>[-0.4, 0.4]	0.0<br>[-0.2, 0.3]	0.0<br>[-0.4, 0.4]	-0.1<br>[-0.6, 0.5]
Effect ratio, %	41.6	63.4	12.3	19.1	NA[a]

RD, risk difference, CI, confidence interval
Suicidal ideation at age 16 was assessed using a current-state item to minimize the risk of reverse causation, which explains the discrepancy between the total effect in this table and the sex difference in Table 2.
Missing data were handled using multiple imputation by chained equations.
Parametric mediational g-formula was used for mediation analysis.
The analysis accounted for sex-mediator and mediator-mediator interactions.
Overall effect was computed as interventional direct effect + interventional indirect effect; difference was computed as total effect – overall effect; effect ratio was computed as (interventional indirect effect + difference) / total effect.
To address mediator-outcome confounding, we adjusted for confounders, including both those unaffected by sex (i.e., age; parental income; living arrangements; neighborhood cohesion; and IQ) and those affected by sex (i.e., body mass index; physical activity; relationships with mother, father, and friends; bullying victimization; physical punishment; problematic internet use; time spent gaming; coffee consumption; pubertal development; belief in equal housework; and each internalizing problem).
Estimates were obtained using MI Boot with pooled samples, based on 20 imputed datasets and 200 bootstrap samples per dataset (4,000 replicates in total); point estimates were calculated as the mean of replicates, and 95% confidence intervals were derived using the percentiles.
[a] Not estimable because the interventional direct effect and the sum of the interventional indirect effect and the difference were in opposite directions.

Supplementary Table 10 | Sensitivity analysis 7: using body dissatisfaction measured using the Stunkard Figure Rating Scale instead of desire for thinness.

Mediators and estimates	Self-report			Parent-report	
	Depressive symptoms	Suicidal ideation	Self-harm	Emotional symptoms	Anxiety
Loneliness and body dissatisfaction					
Total effect<br>RD, % [95% CI]	7.7<br>[5.7, 9.8]	3.1<br>[1.4, 5.1]	3.3<br>[1.8, 4.9]	3.5<br>[1.9, 5.1]	3.1<br>[1.4, 5.3]
Overall effect<br>RD, % [95% CI]	7.7<br>[5.7, 10.1]	3.1<br>[1.4, 4.9]	3.3<br>[1.8, 4.9]	3.6<br>[2.1, 4.9]	3.2<br>[1.5, 5.2]
Interventional direct effect<br>RD, % [95% CI]	4.8<br>[2.2, 7.6]	1.4<br>[-0.3, 3.3]	2.2<br>[0.4, 4.7]	2.9<br>[1.0, 4.9]	4.1<br>[1.9, 7.1]
Interventional indirect effect<br>RD, % [95% CI]	2.9<br>[0.5, 5.2]	1.7<br>[0.4, 2.9]	1.1<br>[-0.3, 2.3]	0.7<br>[-0.6, 2.0]	-0.9<br>[-2.9, 0.7]
Difference<br>RD, % [95% CI]	0.0<br>[-0.5, 0.5]	0.0<br>[-0.4, 0.5]	0.1<br>[-0.2, 0.4]	-0.1<br>[-0.4, 0.4]	-0.1<br>[-0.6, 0.4]
Effect ratio, %	37.5	55.0	33.0	19.4	NA[a]

RD, risk difference, CI, confidence interval
Suicidal ideation at age 16 was assessed using a current-state item to minimize the risk of reverse causation, which explains the discrepancy between the total effect in this table and the sex difference in Table 2.
Missing data were handled using multiple imputation by chained equations.
Parametric mediational g-formula was used for mediation analysis.
The analysis accounted for sex-mediator and mediator-mediator interactions.
Overall effect was computed as interventional direct effect + interventional indirect effect; difference was computed as total effect – overall effect; effect ratio was computed as (interventional indirect effect + difference) / total effect.
To address mediator-outcome confounding, we adjusted for confounders, including both those unaffected by sex (i.e., age; parental income; living arrangements; neighborhood cohesion; and IQ) and those affected by sex (i.e., body mass index; physical activity; relationships with mother, father, and friends; bullying victimization; physical punishment; problematic internet use; time spent gaming; coffee consumption; pubertal development; belief in equal housework; and each internalizing problem).
Estimates were obtained using MI Boot with pooled samples, based on 20 imputed datasets and 200 bootstrap samples per dataset (4,000 replicates in total); point estimates were calculated as the mean of replicates, and 95% confidence intervals were derived using the percentiles.
[a] Not estimable because the interventional direct effect and the sum of the interventional indirect effect and the difference were in opposite directions.

Supplementary Table 11 | Sensitivity analysis 8: including problematic internet use at age 14 as a mediator.

Mediators and estimates	Self-report			Parent-report	
	Depressive symptoms	Suicidal ideation	Self-harm	Emotional symptoms	Anxiety
Loneliness, desire for thinness, and problematic internet use					
Total effect<br>RD, % [95% CI]	7.6<br>[5.1, 10.2]	2.9<br>[1.2, 4.6]	3.3<br>[1.8, 4.8]	3.4<br>[1.9, 5.2]	3.4<br>[1.4, 5.4]
Overall effect<br>RD, % [95% CI]	7.5<br>[5.0, 10.1]	2.8<br>[1.0, 4.5]	3.2<br>[1.8, 4.8]	3.5<br>[1.9, 5.2]	3.5<br>[1.5, 5.4]
Interventional direct effect<br>RD, % [95% CI]	5.3<br>[2.7, 8.2]	1.4<br>[-0.5, 3.3]	2.4<br>[0.7, 4.2]	3.1<br>[1.2, 5.1]	3.8<br>[1.5, 6.3]
Interventional indirect effect<br>RD, % [95% CI]	2.2<br>[0.4, 3.8]	1.4<br>[0.2, 2.6]	0.8<br>[-0.3, 1.8]	0.4<br>[-0.9, 1.6]	-0.4<br>[-1.8, 0.9]
Difference<br>RD, % [95% CI]	0.1<br>[-0.4, 0.6]	0.1<br>[-0.4, 0.5]	0.0<br>[-0.3, 0.3]	0.0<br>[-0.4, 0.4]	-0.1<br>[-0.6, 0.5]
Effect ratio, %	29.5	52.5	26.1	11.1	NA[a]

RD, risk difference, CI, confidence interval
Suicidal ideation at age 16 was assessed using a current-state item to minimize the risk of reverse causation, which explains the discrepancy between the total effect in this table and the sex difference in Table 2.
Missing data were handled using multiple imputation by chained equations.
Parametric mediational g-formula was used for mediation analysis.
The analysis accounted for sex-mediator and mediator-mediator interactions.
Overall effect was computed as interventional direct effect + interventional indirect effect; difference was computed as total effect – overall effect; effect ratio was computed as (interventional indirect effect + difference) / total effect.
To address mediator-outcome confounding, we adjusted for confounders, including both those unaffected by sex (i.e., age; parental income; living arrangements; neighborhood cohesion; and IQ) and those affected by sex (i.e., body mass index; physical activity; relationships with mother, father, and friends; bullying victimization; physical punishment; time spent gaming; coffee consumption; pubertal development; belief in equal housework; and each internalizing problem).
Estimates were obtained using MI Boot with pooled samples, based on 20 imputed datasets and 200 bootstrap samples per dataset (4,000 replicates in total); point estimates were calculated as the mean of replicates, and 95% confidence intervals were derived using the percentiles.
[a] Not estimable because the interventional direct effect and the sum of the interventional indirect effect and the difference were in opposite directions.

Supplementary Table 12 | Sensitivity analysis 9: assuming a joint distribution of mediators (desire for thinness conditional on loneliness).

Mediators and estimates	Self-report			Parent-report	
	Depressive symptoms	Suicidal ideation	Self-harm	Emotional symptoms	Anxiety
Loneliness and desire for thinness					
Total effect<br>RD, % [95% CI]	7.6<br>[5.1, 10.2]	2.8<br>[1.1, 4.5]	3.3<br>[1.8, 4.8]	3.4<br>[1.8, 5.1]	3.5<br>[1.6, 5.4]
Overall effect<br>RD, % [95% CI]	7.6<br>[5.1, 10.1]	2.7<br>[1.1, 4.4]	3.2<br>[1.8, 4.8]	3.4<br>[1.9, 5.1]	3.5<br>[1.6, 5.4]
Interventional direct effect<br>RD, % [95% CI]	5.0<br>[2.5, 7.7]	1.5<br>[-0.2, 3.3]	2.4<br>[0.8, 4.1]	2.6<br>[1.0, 4.5]	3.2<br>[1.0, 5.3]
Interventional indirect effect<br>RD, % [95% CI]	2.6<br>[1.2, 4.1]	1.2<br>[0.1, 2.3]	0.8<br>[-0.1, 1.7]	0.8<br>[-0.2, 1.9]	0.3<br>[-0.8, 1.4]
Difference<br>RD, % [95% CI]	0.0<br>[-0.4, 0.5]	0.1<br>[-0.3, 0.5]	0.1<br>[-0.2, 0.3]	0.0<br>[-0.4, 0.4]	0.0<br>[-0.5, 0.5]
Effect ratio, %	34.3	46.0	26.6	23.3	8.2

RD, risk difference, CI, confidence interval
Suicidal ideation at age 16 was assessed using a current-state item to minimize the risk of reverse causation, which explains the discrepancy between the total effect in this table and the sex difference in Table 2.
Missing data were handled using multiple imputation by chained equations.
Parametric mediational g-formula was used for mediation analysis.
The analysis accounted for sex-mediator and mediator-mediator interactions.
Overall effect was computed as interventional direct effect + interventional indirect effect; difference was computed as total effect – overall effect; effect ratio was computed as (interventional indirect effect + difference) / total effect.
To address mediator-outcome confounding, we adjusted for confounders, including both those unaffected by sex (i.e., age; parental income; living arrangements; neighborhood cohesion; and IQ) and those affected by sex (i.e., body mass index; physical activity; relationships with mother, father, and friends; bullying victimization; physical punishment; problematic internet use; time spent gaming; coffee consumption; pubertal development; belief in equal housework; and each internalizing problem).
Estimates were obtained using MI Boot with pooled samples, based on 20 imputed datasets and 200 bootstrap samples per dataset (4,000 replicates in total); point estimates were calculated as the mean of replicates, and 95% confidence intervals were derived using the percentiles.

Supplementary Table 13 | Sensitivity analysis 10: assuming a joint distribution of mediators (loneliness conditional on desire for thinness).

Mediators and estimates	Self-report			Parent-report	
	Depressive symptoms	Suicidal ideation	Self-harm	Emotional symptoms	Anxiety
Loneliness and desire for thinness					
Total effect<br>RD, % [95% CI]	7.8<br>[5.5, 10.3]	2.9<br>[1.2, 4.7]	3.3<br>[1.8, 4.9]	3.4<br>[1.9, 5.1]	3.5<br>[1.6, 5.5]
Overall effect<br>RD, % [95% CI]	7.8<br>[5.4, 10.3]	2.8<br>[1.2, 4.5]	3.2<br>[1.8, 4.8]	3.4<br>[1.9, 5.1]	3.5<br>[1.7, 5.5]
Interventional direct effect<br>RD, % [95% CI]	5.1<br>[2.5, 7.7]	1.6<br>[-0.2, 3.5]	2.4<br>[0.8, 4.2]	2.6<br>[0.9, 4.4]	3.2<br>[1.1, 5.4]
Interventional indirect effect<br>RD, % [95% CI]	2.7<br>[1.2, 4.3]	1.3<br>[0.2, 2.4]	0.8<br>[-0.1, 1.8]	0.8<br>[-0.2, 1.8]	0.3<br>[-0.8, 1.5]
Difference<br>RD, % [95% CI]	0.0<br>[-0.4, 0.4]	0.1<br>[-0.3, 0.5]	0.1<br>[-0.2, 0.3]	0.0<br>[-0.4, 0.4]	-0.0<br>[-0.5, 0.5]
Effect ratio, %	35.2	46.5	27.5	23.5	8.9

RD, risk difference, CI, confidence interval
Suicidal ideation at age 16 was assessed using a current-state item to minimize the risk of reverse causation, which explains the discrepancy between the total effect in this table and the sex difference in Table 2.
Missing data were handled using multiple imputation by chained equations.
Parametric mediational g-formula was used for mediation analysis.
The analysis accounted for sex-mediator and mediator-mediator interactions.
Overall effect was computed as interventional direct effect + interventional indirect effect; difference was computed as total effect – overall effect; effect ratio was computed as (interventional indirect effect + difference) / total effect.
To address mediator-outcome confounding, we adjusted for confounders, including both those unaffected by sex (i.e., age; parental income; living arrangements; neighborhood cohesion; and IQ) and those affected by sex (i.e., body mass index; physical activity; relationships with mother, father, and friends; bullying victimization; physical punishment; problematic internet use; time spent gaming; coffee consumption; pubertal development; belief in equal housework; and each internalizing problem).
Estimates were obtained using MI Boot with pooled samples, based on 20 imputed datasets and 200 bootstrap samples per dataset (4,000 replicates in total); point estimates were calculated as the mean of replicates, and 95% confidence intervals were derived using the percentiles.

Supplementary Table 14 | Sensitivity analysis 11: using semiparametric generalized additive models to allow for flexible functional forms.

Mediators and estimates	Self-report			Parent-report	
	Depressive symptoms	Suicidal ideation	Self-harm	Emotional symptoms	Anxiety
Loneliness and desire for thinness					
Total effect<br>RD, % [95% CI]	7.7<br>[5.2, 10.1]	2.8<br>[1.2, 4.6]	3.3<br>[1.8, 4.8]	3.4<br>[1.8, 5.1]	3.5<br>[1.6, 5.4]
Overall effect<br>RD, % [95% CI]	7.6<br>[5.1, 10.1]	2.7<br>[1.1, 4.5]	3.2<br>[1.8, 4.7]	3.4<br>[1.8, 5.1]	3.5<br>[1.6, 5.4]
Interventional direct effect<br>RD, % [95% CI]	5.0<br>[2.4, 7.7]	1.5<br>[-0.2, 3.4]	2.4<br>[0.8, 4.2]	2.6<br>[1.0, 4.4]	3.2<br>[1.1, 5.5]
Interventional indirect effect<br>RD, % [95% CI]	2.6<br>[1.1, 4.1]	1.2<br>[0.2, 2.3]	0.8<br>[-0.1, 1.7]	0.8<br>[-0.2, 1.9]	0.3<br>[-0.9, 1.4]
Difference<br>RD, % [95% CI]	0.1<br>[-0.4, 0.5]	0.1<br>[-0.3, 0.5]	0.1<br>[-0.2, 0.3]	0.0<br>[-0.4, 0.4]	0.0<br>[-0.5, 0.5]
Effect ratio, %	34.4	45.9	26.9	23.3	8.0

RD, risk difference, CI, confidence interval
Suicidal ideation at age 16 was assessed using a current-state item to minimize the risk of reverse causation, which explains the discrepancy between the total effect in this table and the sex difference in Table 2.
Missing data were handled using multiple imputation by chained equations.
Mediational g-formula was used for mediation analysis.
The analysis accounted for sex-mediator and mediator-mediator interactions.
Overall effect was computed as interventional direct effect + interventional indirect effect; difference was computed as total effect – overall effect; effect ratio was computed as (interventional indirect effect + difference) / total effect.
To address mediator-outcome confounding, we adjusted for confounders, including both those unaffected by sex (i.e., age; parental income; living arrangements; neighborhood cohesion; and IQ) and those affected by sex (i.e., body mass index; physical activity; relationships with mother, father, and friends; bullying victimization; physical punishment; problematic internet use; time spent gaming; coffee consumption; pubertal development; belief in equal housework; and each internalizing problem).
Estimates were obtained using MI Boot with pooled samples, based on 20 imputed datasets and 200 bootstrap samples per dataset (4,000 replicates in total); point estimates were calculated as the mean of replicates, and 95% confidence intervals were derived using the percentiles.

Supplementary Table 15 | Sensitivity analysis 12: using inverse probability weighting to account for censoring of mediators and outcomes, instead of multiple imputation.

Mediators and estimates	Self-report			Parent-report	
	Depressive symptoms	Suicidal ideation	Self-harm	Emotional symptoms	Anxiety
Loneliness and desire for thinness					
Total effect<br>RD, % [95% CI]	15.1<br>[9.7, 20.8]	6.5<br>[2.5, 9.9]	6.2<br>[2.2, 10.0]	7.4<br>[4.1, 10.8]	3.5<br>[-0.3, 7.0]
Overall effect<br>RD, % [95% CI]	15.1<br>[10.6, 20.6]	6.2<br>[2.3, 9.9]	6.2<br>[2.1, 10.6]	7.4<br>[4.2, 11.4]	3.5<br>[-0.9, 7.0]
Interventional direct effect<br>RD, % [95% CI]	11.4<br>[6.7, 16.9]	4.0<br>[0.3, 8.1]	5.2<br>[1.1, 9.7]	4.3<br>[1.2, 8.0]	2.8<br>[-1.6, 6.8]
Interventional indirect effect<br>RD, % [95% CI]	3.7<br>[-0.3, 6.9]	2.2<br>[-0.4, 5.1]	1.0<br>[-1.4, 3.8]	3.2<br>[0.4, 5.6]	0.7<br>[-1.9, 3.0]
Difference<br>RD, % [95% CI]	0.0<br>[-1.3, 1.3]	0.3<br>[-0.8, 1.3]	0.0<br>[-1.0, 0.8]	-0.1<br>[-1.8, 1.8]	0.0<br>[-1.4, 1.4]
Effect ratio, %	24.4	38.0	16.6	42.1	19.7

RD, risk difference, CI, confidence interval
Suicidal ideation at age 16 was assessed using a current-state item to minimize the risk of reverse causation, which explains the discrepancy between the total effect in this table and the sex difference in Table 2.
Parametric mediational g-formula was used for mediation analysis.
The analysis accounted for sex-mediator and mediator-mediator interactions.
Overall effect was computed as interventional direct effect + interventional indirect effect; difference was computed as total effect – overall effect; effect ratio was computed as (interventional indirect effect + difference) / total effect.
To address mediator-outcome confounding, we adjusted for confounders, including both those unaffected by sex (i.e., age; parental income; living arrangements; neighborhood cohesion; and IQ) and those affected by sex (i.e., body mass index; physical activity; relationships with mother, father, and friends; bullying victimization; physical punishment; problematic internet use; time spent gaming; coffee consumption; pubertal development; belief in equal housework; and each internalizing problem).

Supplementary Table 16 | Sensitivity analysis 13: analyzing depressive, emotional, and anxiety symptoms as continuous variables.

	Self-report		Parent-report
Mediators and estimates	Depressive symptoms	Emotional symptoms	Anxiety symptoms
Loneliness and desire for thinness			
Total effect	1.46	0.49	0.61
β [95% CI]	[1.11, 1.83]	[0.37, 0.62]	[0.36, 0.86]
Overall effect	1.46	0.49	0.61
β [95% CI]	[1.11, 1.82]	[0.37, 0.62]	[0.35, 0.85]
Interventional direct effect	1.02	0.43	0.56
β [95% CI]	[0.66, 1.41]	[0.29, 0.57]	[0.28, 0.83]
Interventional indirect effect	0.44	0.06	0.05
β [95% CI]	[0.22, 0.68]	[-0.01, 0.15]	[-0.10, 0.20]
Difference	0.03	0.00	0.00
β [95% CI]	[-0.07, 0.07]	[-0.05, 0.05]	[-0.11, 0.11]
Effect ratio, %	30.5	13.4	7.9

CI, confidence interval
Missing data were handled using multiple imputation by chained equations.
Parametric mediational g-formula was used for mediation analysis.
The analysis accounted for sex-mediator and mediator-mediator interactions.
Overall effect was computed as interventional direct effect + interventional indirect effect; difference was computed as total effect – overall effect; effect ratio was computed as (interventional indirect effect + difference) / total effect.
To address mediator-outcome confounding, we adjusted for confounders, including both those unaffected by sex (i.e., age; parental income; living arrangements; neighborhood cohesion; and IQ) and those affected by sex (i.e., body mass index; physical activity; relationships with mother, father, and friends; bullying victimization; physical punishment; problematic internet use; time spent gaming; coffee consumption; pubertal development; belief in equal housework; and each internalizing problem).
Estimates were obtained using MI Boot with pooled samples, based on 20 imputed datasets and 200 bootstrap samples per dataset (4,000 replicates in total); point estimates were calculated as the mean of replicates, and 95% confidence intervals were derived using the percentiles.

Supplementary Table 17 | Sensitivity analysis 14: excluding the loneliness item from the Short Mood and Feelings Questionnaire to evaluate depressive symptoms.

	Self-report
Mediators and estimates	Depressive symptoms
Loneliness and desire for thinness	
Total effect<br>*β* [95% CI]	1.37<br>[1.04, 1.70]
Overall effect<br>*β* [95% CI]	1.37<br>[1.04, 1.69]
Interventional direct effect<br>*β* [95% CI]	0.95<br>[0.49, 1.30]
Interventional indirect effect<br>*β* [95% CI]	0.42<br>[0.21, 0.64]
Difference<br>*β* [95% CI]	0.00<br>[-0.06, 0.07]
Effect ratio, %	30.9

CI, confidence interval
Missing data were handled using multiple imputation by chained equations.
Parametric mediational g-formula was used for mediation analysis.
The analysis accounted for sex-mediator and mediator-mediator interactions.
Overall effect was computed as interventional direct effect + interventional indirect effect; difference was computed as total effect – overall effect; effect ratio was computed as (interventional indirect effect + difference) / total effect.
To address mediator-outcome confounding, we adjusted for confounders, including both those unaffected by sex (i.e., age; parental income; living arrangements; neighborhood cohesion; and IQ) and those affected by sex (i.e., body mass index; physical activity; relationships with mother, father, and friends; bullying victimization; physical punishment; problematic internet use; time spent gaming; coffee consumption; pubertal development; belief in equal housework; and depressive symptoms).
Estimates were obtained using MI Boot with pooled samples, based on 20 imputed datasets and 200 bootstrap samples per dataset (4,000 replicates in total); point estimates were calculated as the mean of replicates, and 95% confidence intervals were derived using the percentiles.

Supplementary Table 18 | Sensitivity analysis 15: using an alternative dichotomization cutoff (≥ 7) after excluding the loneliness item from the Short Mood and Feelings Questionnaire to evaluate depressive symptoms.

	Self-report
Mediators and estimates	Depressive symptoms
Loneliness and desire for thinness	
Total effect<br>RD, % [95% CI]	7.9<br>[5.5, 10.6]
Overall effect<br>RD, % [95% CI]	7.8<br>[5.3, 10.7]
Interventional direct effect<br>RD, % [95% CI]	5.0<br>[2.6, 7.9]
Interventional indirect effect<br>RD, % [95% CI]	2.8<br>[1.1, 5.1]
Difference<br>RD, % [95% CI]	0.1<br>[-0.3, 0.5]
Effect ratio, %	37.1

CI, confidence interval
Missing data were handled using multiple imputation by chained equations.
Parametric mediational g-formula was used for mediation analysis.
The analysis accounted for sex-mediator and mediator-mediator interactions.
Overall effect was computed as interventional direct effect + interventional indirect effect; difference was computed as total effect – overall effect; effect ratio was computed as (interventional indirect effect + difference) / total effect.
To address mediator-outcome confounding, we adjusted for confounders, including both those unaffected by sex (i.e., age; parental income; living arrangements; neighborhood cohesion; and IQ) and those affected by sex (i.e., body mass index; physical activity; relationships with mother, father, and friends; bullying victimization; physical punishment; problematic internet use; time spent gaming; coffee consumption; pubertal development; belief in equal housework; and depressive symptoms).
Estimates were obtained using MI Boot with pooled samples, based on 20 imputed datasets and 200 bootstrap samples per dataset (4,000 replicates in total); point estimates were calculated as the mean of replicates, and 95% confidence intervals were derived using the percentiles.

Supplementary Table 19 | Sensitivity analysis 16: using an alternative dichotomization cutoff (≥ 8) after excluding the loneliness item from the Short Mood and Feelings Questionnaire to evaluate depressive symptoms.

	Self-report
Mediators and estimates	Depressive symptoms
Loneliness and desire for thinness	
Total effect<br>RD, % [95% CI]	7.1<br>[4.8, 9.5]
Overall effect<br>RD, % [95% CI]	7.0<br>[4.7, 9.4]
Interventional direct effect<br>RD, % [95% CI]	4.5<br>[2.1, 7.1]
Interventional indirect effect<br>RD, % [95% CI]	2.5<br>[1.1, 4.0]
Difference<br>RD, % [95% CI]	0.1<br>[-0.4, 0.5]
Effect ratio, %	36.4

CI, confidence interval
Missing data were handled using multiple imputation by chained equations.
Parametric mediational g-formula was used for mediation analysis.
The analysis accounted for sex-mediator and mediator-mediator interactions.
Overall effect was computed as interventional direct effect + interventional indirect effect; difference was computed as total effect – overall effect; effect ratio was computed as (interventional indirect effect + difference) / total effect.
To address mediator-outcome confounding, we adjusted for confounders, including both those unaffected by sex (i.e., age; parental income; living arrangements; neighborhood cohesion; and IQ) and those affected by sex (i.e., body mass index; physical activity; relationships with mother, father, and friends; bullying victimization; physical punishment; problematic internet use; time spent gaming; coffee consumption; pubertal development; belief in equal housework; and depressive symptoms).
Estimates were obtained using MI Boot with pooled samples, based on 20 imputed datasets and 200 bootstrap samples per dataset (4,000 replicates in total); point estimates were calculated as the mean of replicates, and 95% confidence intervals were derived using the percentiles.

Supplementary Table 20 | Sensitivity analysis 17: using an alternative dichotomization cutoff (≥ 4) on the Strengths and Difficulties Questionnaire emotional symptoms scale (including the borderline range) to evaluate emotional symptoms.

	Parent-report
Mediators and estimates	Emotional symptoms
Loneliness and desire for thinness	
Total effect<br>RD, % [95% CI]	6.5<br>[4.3, 8.8]
Overall effect<br>RD, % [95% CI]	6.4<br>[4.2, 8.6]
Interventional direct effect<br>RD, % [95% CI]	5.2<br>[2.7, 7.7]
Interventional indirect effect<br>RD, % [95% CI]	1.2<br>[-0.1, 2.7]
Difference<br>RD, % [95% CI]	0.1<br>[-0.5, 0.7]
Effect ratio, %	20.2

CI, confidence interval
Missing data were handled using multiple imputation by chained equations.
Parametric mediational g-formula was used for mediation analysis.
The analysis accounted for sex-mediator and mediator-mediator interactions.
Overall effect was computed as interventional direct effect + interventional indirect effect; difference was computed as total effect – overall effect; effect ratio was computed as (interventional indirect effect + difference) / total effect.
To address mediator-outcome confounding, we adjusted for confounders, including both those unaffected by sex (i.e., age; parental income; living arrangements; neighborhood cohesion; and IQ) and those affected by sex (i.e., body mass index; physical activity; relationships with mother, father, and friends; bullying victimization; physical punishment; problematic internet use; time spent gaming; coffee consumption; pubertal development; belief in equal housework; and emotional symptoms).
Estimates were obtained using MI Boot with pooled samples, based on 20 imputed datasets and 200 bootstrap samples per dataset (4,000 replicates in total); point estimates were calculated as the mean of replicates, and 95% confidence intervals were derived using the percentiles.

Supplementary Table 21 | Sensitivity analysis 18: using a different cutoff (T-score of 60) to evaluate anxiety.

Mediators and estimates	Parent-report<br>Anxiety
Loneliness and desire for thinness	
Total effect<br>RD, % [95% CI]	4.2<br>[1.2, 7.0]
Overall effect<br>RD, % [95% CI]	4.2<br>[1.4, 7.1]
Interventional direct effect<br>RD, % [95% CI]	3.8<br>[0.6, 7.1]
Interventional indirect effect<br>RD, % [95% CI]	0.4<br>[-1.3, 2.0]
Difference<br>RD, % [95% CI]	-0.0<br>[-0.9, 0.9]
Effect ratio, %	8.6

RD, risk difference, CI, confidence interval
Missing data were handled using multiple imputation by chained equations.
Parametric mediational g-formula was used for mediation analysis.
The analysis accounted for sex-mediator and mediator-mediator interactions.
Overall effect was computed as interventional direct effect + interventional indirect effect; difference was computed as total effect – overall effect; effect ratio was computed as (interventional indirect effect + difference) / total effect.
To address mediator-outcome confounding, we adjusted for confounders, including both those unaffected by sex (i.e., age; parental income; living arrangements; neighborhood cohesion; and IQ) and those affected by sex (i.e., body mass index; physical activity; relationships with mother, father, and friends; bullying victimization; physical punishment; problematic internet use; time spent gaming; coffee consumption; pubertal development; belief in equal housework; and anxiety).
Estimates were obtained using MI Boot with pooled samples, based on 20 imputed datasets and 200 bootstrap samples per dataset (4,000 replicates in total); point estimates were calculated as the mean of replicates, and 95% confidence intervals were derived using the percentiles.

Supplementary Table 22 | Sensitivity analysis 19: using a different cutoff (T-score of 70) to evaluate anxiety.

Mediators and estimates	Parent-report<br>Anxiety
Loneliness and desire for thinness	
Total effect<br>RD, % [95% CI]	2.7<br>[1.7, 3.9]
Overall effect<br>RD, % [95% CI]	2.7<br>[1.6, 3.8]
Interventional direct effect<br>RD, % [95% CI]	2.4<br>[1.1, 3.5]
Interventional indirect effect<br>RD, % [95% CI]	0.3<br>[-0.6, 1.2]
Difference<br>RD, % [95% CI]	0.0<br>[-0.4, 0.3]
Effect ratio, %	11.3

RD, risk difference, CI, confidence interval
Missing data were handled using multiple imputation by chained equations.
Parametric mediational g-formula was used for mediation analysis.
The analysis accounted for sex-mediator and mediator-mediator interactions.
Overall effect was computed as interventional direct effect + interventional indirect effect; difference was computed as total effect – overall effect; effect ratio was computed as (interventional indirect effect + difference) / total effect.
To address mediator-outcome confounding, we adjusted for confounders, including both those unaffected by sex (i.e., age; parental income; living arrangements; neighborhood cohesion; and IQ) and those affected by sex (i.e., body mass index; physical activity; relationships with mother, father, and friends; bullying victimization; physical punishment; problematic internet use; time spent gaming; coffee consumption; pubertal development; belief in equal housework; and anxiety).
Estimates were obtained using MI Boot with pooled samples, based on 20 imputed datasets and 200 bootstrap samples per dataset (4,000 replicates in total); point estimates were calculated as the mean of replicates, and 95% confidence intervals were derived using the percentiles.

Supplementary Table 23 | Covariate distributions by sex and loneliness at age 14:

Variables	Boys without loneliness	Boys with loneliness	Girls without loneliness	Girls with loneliness
Age (years), range	11.6–13.0	11.7–12.8	11.6–12.9	11.6–12.9
Parental income, JPY, %				
< 5,000,000	18.3	25.7	14.4	20.8
5,000,000 to 9,999,999	51.9	48.6	54.6	57.7
≥ 10,000,000	29.9	25.7	31.0	21.5
Living arrangements, %				
With father	92.7	87.3	93.3	92.9
Separated from father	7.3	12.7	6.7	7.1
Neighborhood cohesion, range	0.0–20.0	7.0–20.0	0.0–20.0	6.0–20.0
IQ, range	72.5–149.7	79.7–148.2	51.8–152.3	72.4–141.3
Body mass index, range	11.6–31.2	13.8–26.5	12.9–29.0	13.3–27.4
Physical activity, %				
Once per month or more	88.5	89.5	82.4	81.2
Less than once per month	11.5	10.5	17.6	18.8
Relationship with mother, range	0.0–5.0	1.6–5.0	0.0–5.0	0.0–5.0
Relationship with father, range	0.0–5.0	0.0–5.0	0.0–5.0	0.0–5.0
Relationships with friends, range	0.0–5.0	1.6–5.0	0.1–5.0	1.0–5.0
Bullying victimization, %				
No	82.0	75.0	90.3	83.4
Yes or somewhat yes	18.0	25.0	9.7	16.6
Physical punishment, %				
Never or rarely	63.5	60.3	71.3	69.5
Sometimes, often, or always	36.5	39.7	28.7	30.5
Problematic internet use, range	0.0–20.0	0.0–17.0	0.0–18.0	0.0–16.0
Time spent gaming, %				
Less than one hour per day	61.0	56.2	86.6	80.0
One hour per day or longer	39.0	43.8	13.4	20.0
Coffee consumption, %				
None	91.6	93.8	91.2	91.6
One cup per day or more	8.4	6.2	8.8	8.4
Pubertal development, %				
Tanner stage 1	15.3	15.3	15.1	12.0
Tanner stages 2–5	84.7	84.7	84.9	88.0
Belief in equal housework, %				
Strongly agree or agree	62.4	56.6	64.9	72.5
Disagree or strongly disagree	37.6	43.4	35.1	27.5
Depressive symptoms, %				
No	84.4	66.2	84.2	69.1
Yes	15.6	33.8	15.8	30.9
Suicidal ideation, %				
No	82.1	61.1	81.4	65.6
Yes	17.9	38.9	18.6	34.4
Self-harm, %				
No	90.5	86.4	88.1	87.3
Yes	9.5	13.6	11.9	12.7
Emotional symptoms, %				
No	94.8	92.4	95.3	88.3
Yes	5.2	7.6	4.7	11.7
Anxiety, %				
No	89.7	89.6	91.8	86.7
Yes	10.3	10.4	8.2	13.3

Supplementary Table 24 | Covariate distributions by sex and desire for thinness at age 14:

Variables	Boys without desire for thinness	Boys with desire for thinness	Girls without desire for thinness	Girls with desire for thinness
Age (years), range	11.6–13.0	11.7–12.9	11.6–12.9	11.6–12.9
Parental income, JPY, %				
< 5,000,000	18.5	20.9	14.7	16.6
5,000,000 to 9,999,999	52.0	49.1	53.2	57.6
≥ 10,000,000	29.6	30.0	32.1	25.7
Living arrangements, %				
With father	93.0	86.8	93.6	92.8
Separated from father	7.0	13.2	6.4	7.2
Neighborhood cohesion, range	0.0–20.0	4.0–20.0	0.0–20.0	1.0–20.0
IQ, range	72.5–149.7	75.0–143.3	51.8–148.9	67.3–152.3
Body mass index, range	11.6–28.4	11.8–31.2	12.9–24.8	14.1–29.0
Physical activity, %				
Once per month or more	89.2	83.5	82.4	82.2
Less than once per month	10.8	16.5	17.6	17.8
Relationship with mother, range	0.0–5.0	0.4–5.0	0.0–5.0	0.0–5.0
Relationship with father, range	0.0–5.0	0.0–5.0	0.0–5.0	0.0–5.0
Relationships with friends, range	0.0–5.0	1.1–5.0	0.4–5.0	0.1–5.0
Bullying victimization, %				
No	82.7	73.4	90.1	87.8
Yes or somewhat yes	17.3	26.6	9.9	12.2
Physical punishment, %				
Never or rarely	63.7	61.7	72.3	69.3
Sometimes, often, or always	36.3	38.3	27.7	30.7
Problematic internet use, range	0.0–20.0	0.0–17.0	0.0–18.0	0.0–18.0
Time spent gaming, %				
Less than one hour per day	61.9	52.0	86.4	84.4
One hour per day or longer	38.1	48.0	13.6	15.6
Coffee consumption, %				
None	92.3	89.3	92.2	90.3
One cup per day or more	7.7	10.7	7.8	9.7
Pubertal development, %				
Tanner stage 1	14.6	20.8	18.1	10.1
Tanner stages 2–5	85.4	79.2	81.9	89.9
Belief in equal housework, %				
Strongly agree or agree	61.9	62.8	66.0	66.7
Disagree or strongly disagree	38.1	37.2	34.0	33.3
Depressive symptoms, %				
No	84.4	72.1	84.1	78.2
Yes	15.6	27.9	15.9	21.8
Suicidal ideation, %				
No	80.9	76.6	82.2	73.8
Yes	19.1	23.4	17.8	26.2
Self-harm, %				
No	90.4	87.0	87.5	88.6
Yes	9.6	13.0	12.5	11.4
Emotional symptoms, %				
No	95.3	88.7	94.6	93.3
Yes	4.7	11.3	5.4	6.7
Anxiety, %				
No	90.1	85.8	91.8	89.8
Yes	9.9	14.2	8.2	10.2

Supplementary Table 25 | Positivity assessment for desire for thinness and loneliness. Because sex was assigned at birth, the exposure positivity condition was assessed without conditioning on covariates[1] and was satisfied by the presence of both girls and boys in the analytic sample: Pr(Girl = 1) = 0.47 and Pr(Girl = 0) = 0.53. The table presents mediator positivity assessments, for which probabilities were estimated conditional on covariates and sex. Covariates included each pre-mediator outcome.

Outcome	Variable	Category	Probability assessed	Minimum estimated probability across datasets
Depressive symptoms	Desire for thinness	No	Pr(Desire for thinness = 0 | Covariates, Sex)	0.01
		Yes	Pr(Desire for thinness = 1 | Covariates, Sex)	0.01
	Loneliness	No	Pr(Loneliness = 0 | Covariates, Sex)	0.45
		Yes	Pr(Loneliness = 1 | Covariates, Sex)	0.02
Suicidal ideation	Desire for thinness	No	Pr(Desire for thinness = 0 | Covariates, Sex)	0.01
		Yes	Pr(Desire for thinness = 1 | Covariates, Sex)	0.01
	Loneliness	No	Pr(Loneliness = 0 | Covariates, Sex)	0.42
		Yes	Pr(Loneliness = 1 | Covariates, Sex)	0.01
Self-harm	Desire for thinness	No	Pr(Desire for thinness = 0 | Covariates, Sex)	0.01
		Yes	Pr(Desire for thinness = 1 | Covariates, Sex)	0.01
	Loneliness	No	Pr(Loneliness = 0 | Covariates, Sex)	0.50
		Yes	Pr(Loneliness = 1 | Covariates, Sex)	0.02
Emotional symptoms	Desire for thinness	No	Pr(Desire for thinness = 0 | Covariates, Sex)	0.01
		Yes	Pr(Desire for thinness = 1 | Covariates, Sex)	0.01
	Loneliness	No	Pr(Loneliness = 0 | Covariates, Sex)	0.44
		Yes	Pr(Loneliness = 1 | Covariates, Sex)	0.02
Anxiety	Desire for thinness	No	Pr(Desire for thinness = 0 | Covariates, Sex)	0.01
		Yes	Pr(Desire for thinness = 1 | Covariates, Sex)	0.01
	Loneliness	No	Pr(Loneliness = 0 | Covariates, Sex)	0.50
		Yes	Pr(Loneliness = 1 | Covariates, Sex)	0.02

Reference

1. Nguyen, T. Q., Schmid, I., Ogburn, E. L. & Stuart, E. A. Clarifying causal mediation analysis: Effect identification via three assumptions and five potential outcomes. J. Causal Inference 10, 246–279 (2022).

Supplementary Table 26 | Attrition from age 12 to age 16 stratified by sex and baseline internalizing symptomatology

Sex	Baseline internalizing symptomatology	N	Retained, n (%)	Attrited, n (%)
Boys	Absent	1093	844 (77.2)	249 (22.8)
	Present	577	477 (82.7)	100 (17.3)
Girls	Absent	932	735 (78.9)	197 (21.1)
	Present	545	436 (80.0)	109 (20.0)

Twenty-four participants were excluded from this table because all five internalizing problem measures at age 12 were missing, precluding classification of baseline internalizing symptomatology.
